# *IDA* (*INFLORESCENCE DEFICIENT IN ABSCISSION*)-like peptides and *HAE* (*HAESA*)-like receptors regulate corolla abscission in *Nicotiana benthamiana* flowers

**DOI:** 10.1186/s12870-021-02994-8

**Published:** 2021-05-21

**Authors:** Daniel Ventimilla, Karelia Velázquez, Susana Ruiz-Ruiz, Javier Terol, Miguel A. Pérez-Amador, Mª. Carmen Vives, José Guerri, Manuel Talon, Francisco R. Tadeo

**Affiliations:** 1 Centro de Genómica - Instituto Valenciano de Investigaciones Agrarias (IVIA), Moncada, 46113 Valencia, Spain; 2 Centro de Protección Vegetal y Biotecnología, Instituto Valenciano de Investigaciones Agrarias (IVIA), Moncada, 46113 Valencia, Spain; 3 Instituto de Biología Molecular y Celular de Plantas (IBMCP), CSIC-Universidad Politécnica de Valencia. CPI Ed. 8E, Camino de Vera s/n, 46022 Valencia, Spain

**Keywords:** Abscission zone, Abscission signaling module, Cell separation, Gene silencing and overexpression, Hormone peptide, LRR-RLKs, Solanaceae

## Abstract

**Background:**

Abscission is an active, organized, and highly coordinated cell separation process enabling the detachment of aerial organs through the modification of cell-to-cell adhesion and breakdown of cell walls at specific sites on the plant body known as abscission zones. In *Arabidopsis thaliana*, abscission of floral organs and cauline leaves is regulated by the interaction of the hormonal peptide INFLORESCENCE DEFICIENT IN ABSCISSION (IDA), a pair of redundant receptor-like protein kinases, HAESA (HAE) and HAESA-LIKE2 (HSL2), and SOMATIC EMBRYOGENESIS RECEPTOR-LIKE KINASE (SERK) co-receptors. However, the functionality of this abscission signaling module has not yet been demonstrated in other plant species.

**Results:**

The expression of the pair of *NbenIDA1* homeologs and the receptor *NbenHAE.1* was supressed at the base of the corolla tube by the inoculation of two virus-induced gene silencing (VIGS) constructs in *Nicotiana benthamiana*. These gene suppression events arrested corolla abscission but did not produce any obvious effect on plant growth. VIGS plants retained a higher number of corollas attached to the flowers than control plants, an observation related to a greater corolla breakstrength. The arrest of corolla abscission was associated with the preservation of the parenchyma tissue at the base of the corolla tube that, in contrast, was virtually collapsed in normal corollas. In contrast, the inoculation of a viral vector construct that increased the expression of *NbenIDA1A* at the base of the corolla tube negatively affected the growth of the inoculated plants accelerating the timing of both corolla senescence and abscission. However, the heterologous ectopic overexpression of citrus *CitIDA3* and Arabidopsis *AtIDA* in *N. benthamiana* did not alter the standard plant phenotype suggesting that the proteolytic processing machinery was unable to yield active peptides.

**Conclusion:**

Here, we demonstrate that the pair of *NbenIDA1* homeologs encoding small peptides of the *IDA*-like family and the receptor *NbenHAE.1* control cellular breakdown at the base of the corolla tube awhere an adventitious AZ should be formed and, therefore, corolla abscission in *N. benthamiana* flowers. Altogether, our results provide the first evidence supporting the notion that the IDA-HAE/HSL2 signaling module is conserved in angiosperms.

**Supplementary Information:**

The online version contains supplementary material available at 10.1186/s12870-021-02994-8.

## Background

Abscission is a fundamental cell separation process in plant biology that accounts for a highly beneficial evolutionary adaptation for plants: the discarding of infected, senescent or physiologically damaged organs and the highly efficient seed dispersal [1]. However, from an agricultural point of view, abscission has a huge impact on yield, leading to high production losses. Therefore, the greater the knowledge about the control of the abscission process, the more efficient the cultivation of species of agronomic interest will be.

Abscission of floral organs and cauline leaves in *Arabidopsis thaliana* (from now on, Arabidopsis) is regulated by the interaction of the hormone peptide INFLORESCENCE DEFICIENT IN ABSCISSION (IDA), the two redundant receptor-like protein kinases HAESA (HAE) and HAESA-LIKE2 (HSL2), and the SOMATIC EMBRYOGENESIS RECEPTOR-LIKE KINASE (SERK) co-receptors [2, 3]. Once a stable complex is formed between the IDA peptide and HAE-like/SERK heterodimers, the kinase domains transphosphorylate each other and relay the signal to a mitogen-activated protein kinase (MAPK) cascade [4, 5]. It was determined that the MAPK cascade inhibits the activity of the KNOTTED-LIKE HOMEOBOX (KNOX) transcription factor BREVIPEDICELLUS (BP)/KNOTTED-LIKE FROM ARABIDOPSIS THALIANA 1 (KNAT1), which in turn de-represses other KNOX genes (*KNAT2* and *KNAT6*) to induce the expression of a set of cell wall remodeling enzymes and modifying proteins that allow floral organ abscission [6, 7]. In addition to Arabidopsis, *IDA*-like genes have also been identified in a number of crop species. Thus, it has been reported that specific *IDA*-like genes were highly expressed in abscission zones (AZs) in tomato, soybean, oil palm, citrus, litchi or yellow lupine and also at the base of the corolla tube of *Nicotiana benthamiana* flowers during abscission [8–13]. These observations strongly suggest that *IDA*-like genes might conserve in other species the same function that *IDA* exerts in Arabidopsis regulating cell separation during organ abscission. It has been also shown that synthetic IDA peptides were able to induce early floral organ abscission in Arabidopsis [14] and abscission of flowers, mature fruits and leaves in yellow lupine, oil palm and Poplar, respectively [12, 15]. Additionally, *IDA* homologues of citrus (*CitIDA3*) and litchi (*LcIDA1*) expressed in Arabidopsis were functional producing earlier floral organ abscission and rescuing the *ida2* abscission deficiency [10, 11]. Similarly, the ectopic overexpression of a *HAE*-like homolog of litchi, *LcHSL2*, completely rescued abscission of floral organs in the Arabidopsis double mutant *hae/hsl2* [16]. Finally, the ectopic expression of *LcKNAT1*, the litchi homolog of Arabidopsis *BP*/*KNAT1*, prevented the abscission of flowers and floral organs in tomato and Arabidopsis, respectively [17]. Despite the high number of results pointing to the conservation of the IDA-HAE/HSL2 signaling module in various angiosperms, there is still some reluctance in the scientific community to generalize its function to other plant species [18]. Therefore, it would be advisable to provide unequivocal demonstration of its functionality in plant species other than Arabidopsis to address the doubts and objections that still remain as related to the conservation of the IDA-HAE/HSL2 abscission signaling module.

In the last two decades, viral vectors have been used as an efficient tool to elucidate the function of many genes using virus induced gene silencing (VIGS) or to express valuable proteins involved in a wide range of plant development processes, including organ abscission. For instance, the importance of polygalacturonases (PGs) in tomato leaf abscission was demonstrated by VIGS approach using *Tobacco rattle virus* (*TRV*)-based vectors [19], as PGs participate in the dissolution of the middle lamella in AZs of different aerial organs of tomato plants (for a review, see [20]). Also using tomato as plant material and *TRV*-based vectors, it was shown that the silencing of *SlPIN1* accelerated flower abscission by increasing auxin accumulation in the ovary and decreasing the auxin content in the petiole AZ [21]. The downregulation by VIGS of auxin conjugate hydrolases *SlILL1*, *SlILL5*, and *SlILL6* significantly reduced auxin concentration in pedicel AZs increasing flower abscission rate [22].

VIGS has also been applied in the study of petal abscission. The role of an auxin/indole-3-acetic acid (Aux/IAA) transcription repressor and two ethylene response factors (ERFs) during petal abscission in hybrid tea rose (*Rosa hybrida*) was evidenced by VIGS using *TRV*-based vectors as well [23]. Up-regulation of six *Aux/IAA* genes was detected in rose petal AZs during petal shedding and the silencing of one of these up-regulated *Aux/IAA* genes, *RhIAA16*, by VIGS accelerated petal abscission suggesting that transcription repression by Aux/IAA proteins in petal AZs might be required to prevent premature abscission. It was also shown that the expression level of two ERFs, *RhERF1* and *RhERF4*, was regulated by ethylene and auxin, respectively, in rose petal AZs during petal shedding [24]. Treatment of rose flowers with ethylene reduced the expression of *RhERF1*, while the expression of *RhERF4* was significantly induced in petal AZs by auxin. VIGS silencing of both rose ERFs accelerates rose petal abscission, a process related to the reduction of pectic galactan in the rose petal AZ associated with the expression level of the β-galactosidase *RhBGLA1* [24].

Transient expression studies and VIGS have been shown to be particularly feasible in *N. benthamiana* in order to conduct functional studies. Actually, *N. benthamiana* is one of the most commonly used model plant organisms to perform host-pathogen interaction studies due to its hypersensitivity to viruses and other pathogenic agents [25]. It has been also shown that *Citrus leaf blotch virus* (*CLBV*)-based viral vectors are able to either silencing genes (*clbv3’* vector) or expressing proteins (*clbv3’pr* vector) both in citrus and in *N. benthamiana* plants [26–29]. The *CLBV* virus is not limited to the phloem and therefore reaches and accumulates in meristems and vegetative and reproductive organs [30]. In fact, Green fluorescent protein (GFP) detection in corolla limb lobes of flowers from *N. benthamiana* plants inoculated with the construct *clbv3’pre*-GFP infective clone demonstrated that these vectors are also effective in reproductive tissues [30].

In this survey, a strategy based on *CLBV* VIGS vectors was used to characterize the regulatory role of the pair of *NbenIDA1* homeologs and its potential receptor kinases of the *HAE*-like family in corolla abscission of *N. benthamina* flowers.

## Results

### Silencing and overexpression of *Nicotiana benthamiana IDA*-like and *HAE*-like genes using a viral vector based on *Citrus leaf blotch virus*

In a previous study, we found that the expression pattern of both the pair of *NbenIDA1* and *NbenHAE* homeologs paralleled the corolla abscission process in *N. benthamiana* [13]. The C-terminal proline-rich signature of the *N. benthamiana* IDA-like peptides, the so-called PIP domain [31], of the pair of NbenIDA1 peptides, NbenIDA1A and NbenIDA1B, conserved the amino acid residues Ser62, Pro64, Ser65, and Asn69 that were demonstrated to be essential in the interaction of Arabidopsis AtIDA with the peptide binding pocket of the AtHAE receptor [5] (Additional file 1). Regarding the amino acid residues inside the peptide binding pocket of HAE that are key to the interaction with IDA [5], the pair of NbenHAE receptors, NbenHAE.1 and NbenHAE.2, also conserved the five critical amino acid residues for ligand-receptor binding (Glu266, Phe289, Ser311, Arg407 and Arg409) and others with a secondary role (Additional file 1). Taken together, these observations suggest that the pair of NbenHAE homeolog receptors and the pair of NbenIDA1 homeolog peptides might conform a functional signaling module in corolla abscission of *N. benthamiana* flowers. In order to explore this possibility, we generated constructs of the *CLBV*-based vectors for the silencing of either *NbenIDA1* or *NbenHAE* homeologs (*clbv3′*-NbenIDA1 and *clbv3’*-NbenHAE constructs, respectively) by selecting silencing triggering sequences common to both pairs of homeologs (Additional file 2). Furthermore, we generated additional constructs to investigate the effect of the ectopic expression of *NbenIDA1A* (*clbv3’pr*-NbenIDA1 construct) and other *IDA*-like genes such as the *CitIDA3* gene from *Citrus* and the *AtIDA* gene from Arabidopsis (*clbv3’pr*-CitIDA3 and *clbv3’pr*-AtIDA constructs, respectively), in *N. benthamiana* plants (Additional file 2).

### The inoculation of *clbv3’*-NbenIDA and *clbv3’*-NbenHAE constructs arrest corolla abscission

**Fig. 1:**
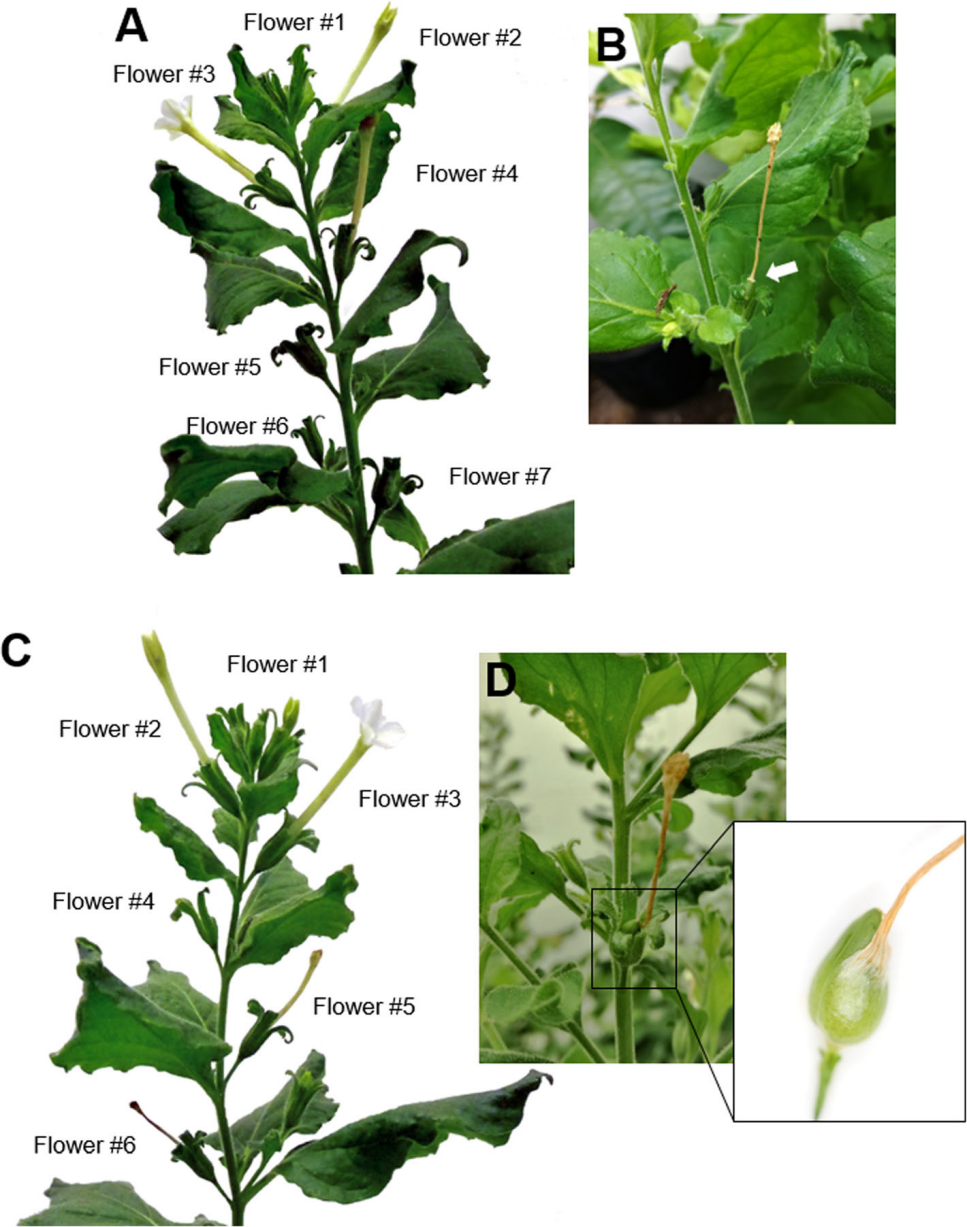
Morphological phenotypes of *N. benthamiana* plants, 4 weeks post inoculation with the control vector *clbv3’* (**a**) and *clbv3’*-NbenIDA construct (**c**). In plants inoculated with the *clbv3’* vector, the base of the necrotic corolla tubes has completely disappeared in advanced stages of flower development. In these plants, the necrotic corolla tubes are only weakly held by the pointed apical end of the capsule (**b**). In plants inoculated with *clbv3’*-NbenIDA construct, necrotic corolla tubes remain attached to flowers (**d**). This feature is clearly distinguishable in a close-up of a silenced flower in which the sepals have been removed showing that the necrotic corolla tube is still attached to the receptacle. The morphological phenotype of plants inoculated with the *clbv3’*-NbenHAE construct was identical to those plants inoculated with the *clbv3’*-NbenIDA construct

At the morphological level, the inoculation of *clbv3’*-NbenIDA and *clbv3’*-NbenHAE constructs did not produce any obvious effect on plant growth, either affected the rate of development or the size of the major vegetative or reproductive organs of the inoculated plants (Fig. 1a and c and Additional file 3). However, although plants inoculated with either constructs grew and developed normally just as controls, it was conspicuous that corolla tubes remained attached to the flower receptacles (Fig. 1d and Additional file 3). The close-up of a silenced flower from which the ring of sepals (calyx) has been removed shows that the necrotic corolla tube was still attached to the receptacle. This observation is rather relevant since in control plants the base of the corolla tube is in the process of disappearing in advanced stages of flower development (Fig. 1a, flowers #5 to #7) and the remaining senescent corolla is weakly attached to the apical pointed end of the fruit, called capsule in the solanaceae subfamily Nicotianoideae (Fig. 1b). To identify unequivocally the homeologs that were silenced by the VIGS constructs, we carried out RNA-seq analysis on *N. benthamiana* corolla bases at flower developmental stage 4 from control (CLBV) and silencing construct (IDAsil, and HAEsil) inoculated plants (Additional file 4). As expected, the inoculation of the *clbv3’*-NbenIDA silencing construct resulted in the suppression of the pair of *NbenIDA1* homeologs at the base of the corolla tube whereas the *clbv3’*-NbenHAE silencing construct only suppressed the expression of *NbenHAE.1*, but not that of *NbenHAE.2*.

**Fig. 2:**
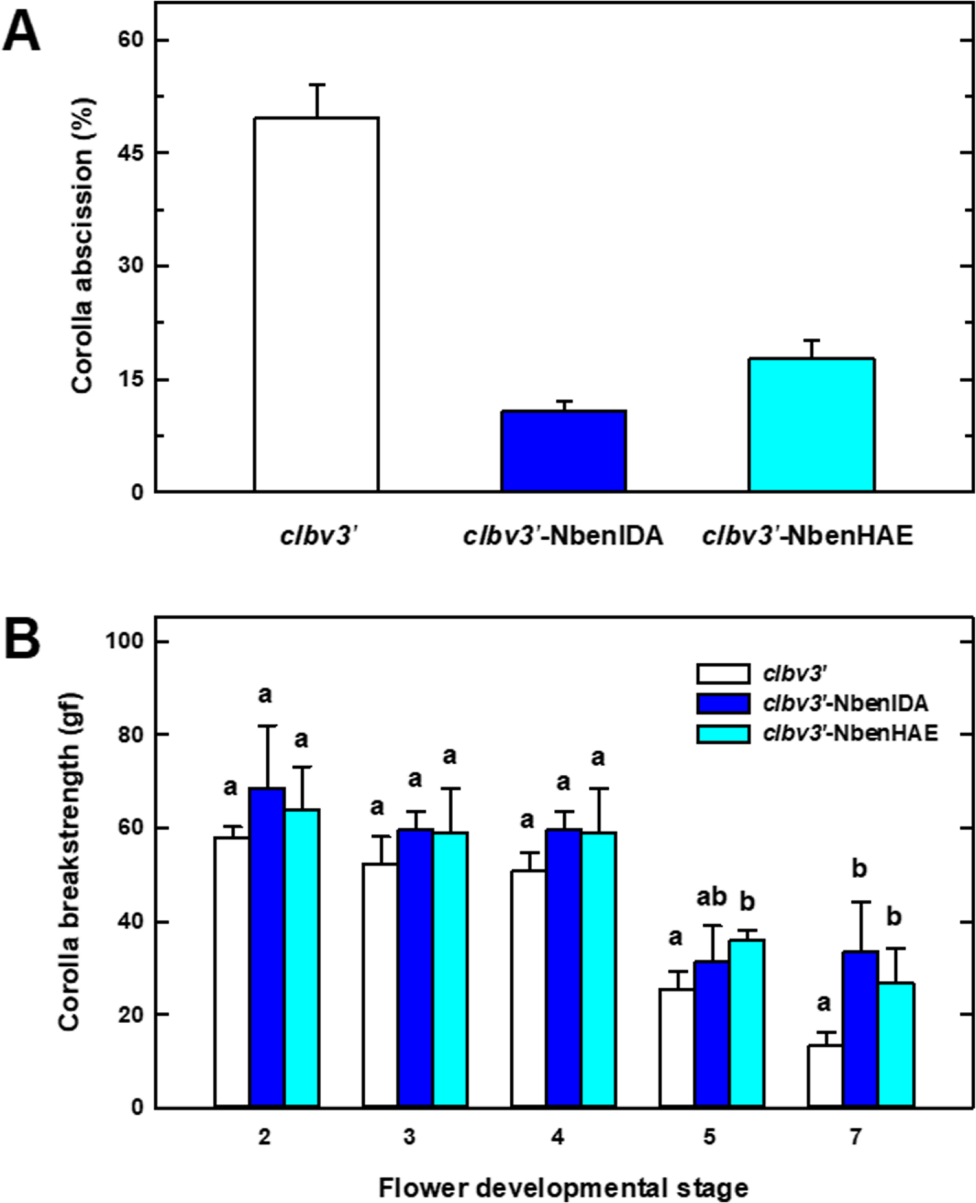
Corolla attachment in plants inoculated with the control vector *clbv3’* and the silencing *clbv3’*-NbenIDA and *clbv3’*-NbenHAE constructs showing the percentage of corollas shed after stage 7 of flower development (**a**) and the force required to remove the flower corolla (corolla breakstrength; cBS) (**b**). All results are means of 4 silencing experiments involving 6 plants per inoculation (≥ 40 measurements at each flower developmental stage) ± standard error. Different letters in Fig. 2b indicate significant differences between VIGS constructs (Student’s t-test, *P* < 0.05)

Natural shedding of *N. benthamiana* corollas takes place after stage 7 of flower development, coinciding with the completion of the senescence process of the corolla (Additional file 5). Quantitation of the number of retained/abscised corollas in plants inoculated with the silencing constructs *clbv3’-*NbenIDA or *clbv3’-*NbenHAE revealed that they retained a higher number of corollas attached to the flowers after the developmental stage 7 than control plants. Among the two groups of silenced plants, the *clbv3’*-NbenIDA construct produced a slightly higher percentage of retention (Fig. 2a). At this flower stage, approximately 50% of corollas were detached in control plants, while total abscission of corollas was 10 and 18%, respectively, in plants inoculated with *clbv3’-*NbenIDA and *clbv3’-*NbenHAE constructs.

In order to determine the force required to remove flower corollas in *N. benthamiana* plants, corolla breakstrength (cBS) was measured using a dynamometer at different flower developmental stages (Fig. 2b). Control plants exhibited a gradual decline of cBS values in flowers at stages 2 to 7. Both control and silenced plants required a similar amount of force to detach corollas in flowers between stages 2 to 4. In contrast, control plants required lower force values to detach the corollas in comparison with silenced plants at flower stages 5 and 7. cBS measurements in plants inoculated with *clbv3’*-NbenIDA1 and *clbv3’*-NbenHAE silencing constructs remained stable during stage 5 to 7 (Fig. 2b). At flower stage 7, corolla detachment in control plants was reached with forces as weak as about 10 gf, while in *clbv3’-*NbenIDA and *clbv3’-*NbenHAE inoculated plants higher forces, about 40 and 30 gf, respectively, were required. This feature should be associated with the preservation of the attachment of the base of the corolla tube to the flower receptacle and the arrest of corolla abscission observed in silenced plants (see Fig. 1c and d).

### Anatomy at the base of the corolla tube

**Fig. 3:**
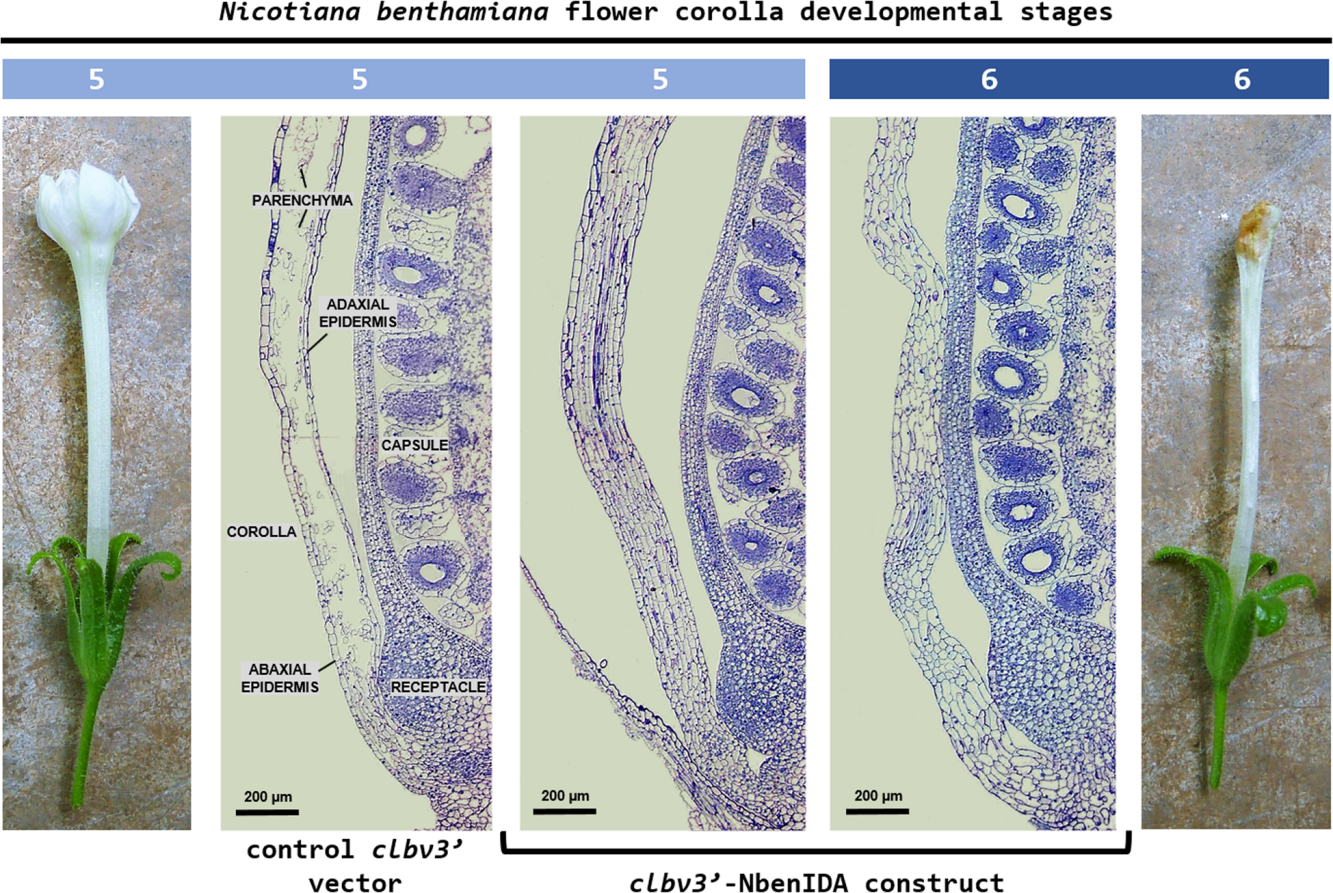
Anatomical comparisons at the base of the corolla tube between flowers of *N. benthamiana* plants inoculated with the control vector *clbv3’* and the *clbv3’*-NbenIDA construct

Next, we studied the histological changes at the base of the corolla tube in flowers from control and VIGS plants. The light microscopic inspection of control flowers at developmental stage 5 revealed the collapse of the parenchyma tissue over a wide area at the base of the corolla tube probably due to the action of cell wall hydrolytic enzymes (Fig. 3). In addition, the walls of the adaxial and abaxial epidermis of the corolla showed no signs of structural damage suggesting that they should be protected to some extent from hydrolytic enzyme activity.

The anatomical structure at the base of the corolla tube of flowers in which the expression of the pair of *NbenIDA1* homeologs was suppressed (plants inoculated with the *clbv3’*-NbenIDA construct) showed different aspect from that observed in control flowers. Cell wall breakdown observed at flower development stage 5 in the parenchyma tissue of control corollas was completely arrested in *NbenIDA1*-silenced flowers (Fig. 3). Parenchyma tissue cells retained cellular integrity in the base of the corolla tube at flower development stage 6 despite the wavy shape of the corolla. Moreover, whereas corolla senescence in *N. benthamiana* flowers is characterized by a gradual loss of turgor (Additional file 5), the suppression of the pair of *NbenIDA1* homeologs did not appear to modify this process. The enlargement of the capsule contributes to the disintegration of the base of the senescent corolla tube and therefore to its detachment from the flower receptacle (Additional file 5). The force with which the corolla withstands the enlargement of the capsule must apparently be related both to the maintenance of the anatomical structure and to the loss of cell wall elasticity and cell turgor. Thus, the minor difference in cBS recorded between control corollas and those from silenced plants (see Fig. 2b) might only be associated with the maintenance of the anatomical structure at the base of the corolla tube.

### Overexpression of *NbenIDA1A* decreases plant growth and accelerates corolla senescence and abscission

A *CLBV*-based expression vector (*clbv3’pr*) containing an additional sgRNA promoter for stable and high-level expression [26] was used to study the effect of increased transcript levels of the endogenous *NbenIDA1A* gene on *N. benthamiana* plants (*clbv3’pr*-NbenIDA1 construct) and also the heterologous expression of foreign *IDA*-like genes from Arabidopsis and citrus (*clbv3’pr*-AtIDA and *clbv3’pr*-CitIDA3 constructs, respectively) (Additional file 2).

**Fig. 4:**
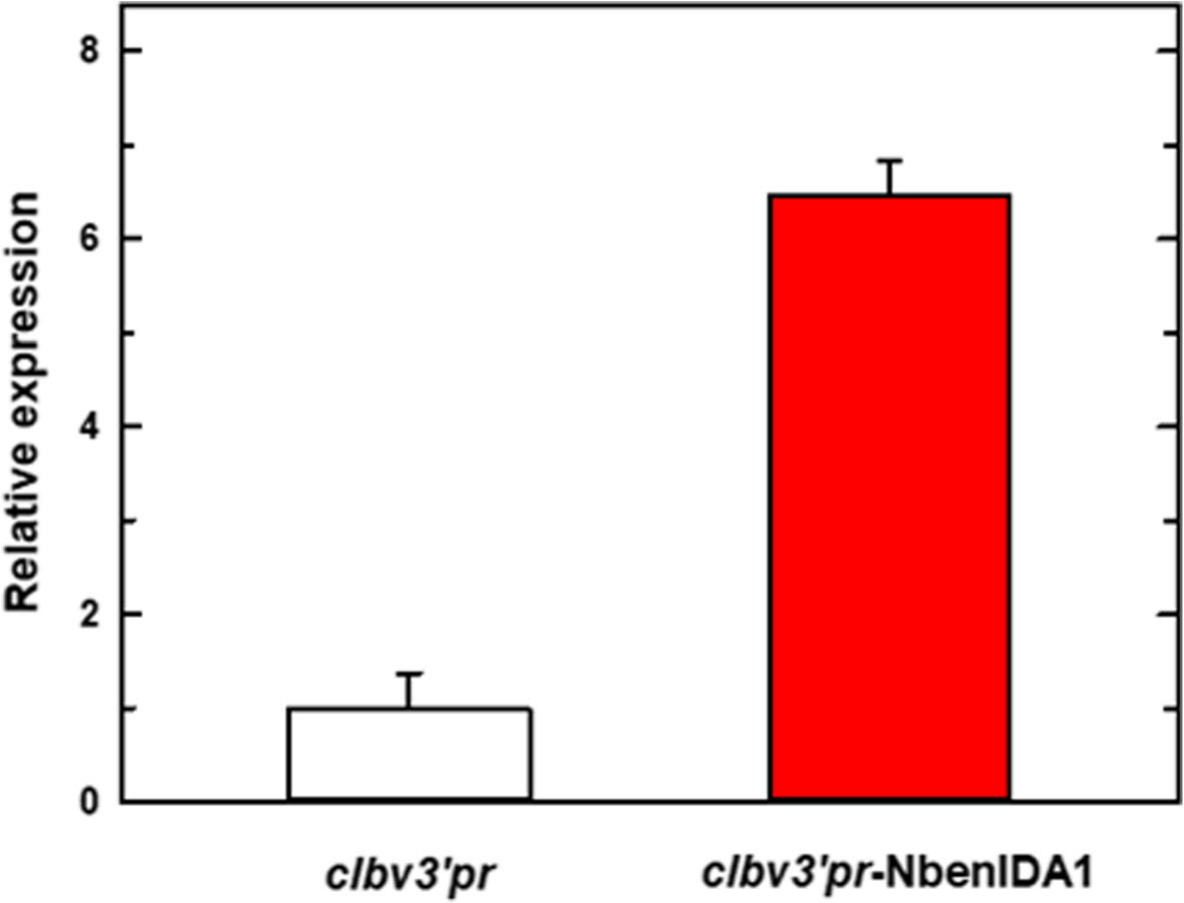
Relative expression levels of *NbenIDA1A* in the corolla base of flowers in stage 2 in *N. benthamiana* plants inoculated with the control vector *clbv3’pr* and the *CLBV*-based expression construct *clbv3’pr*-NbenIDA1. Relative expression levels correspond to mean values of four samples from six independent agro-inoculated plants

Regarding plants inoculated with the *clbv3’pr*-NbenIDA1 construct, the expression level of *NbenIDA1A* in the corolla base of flowers at developmental stage 2 was more than six times higher than that in control flowers (Fig. 4). Therefore, the phenotype of plants inoculated with the *CLBV* expression vector *clbv3’pr*-NbenIDA1 should be related to the over-accumulation of *NbenIDA1A* transcripts.

**Fig. 5:**
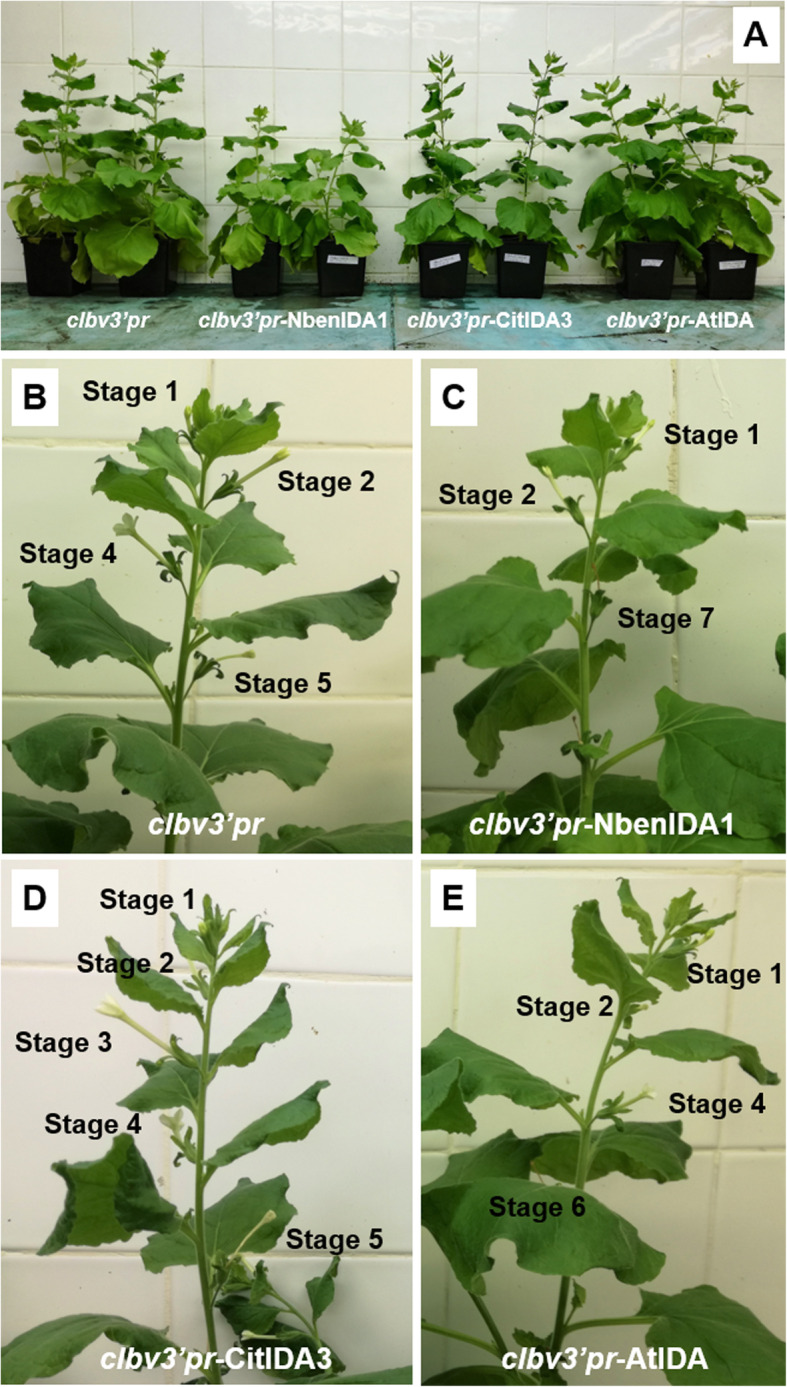
Virus induced gene expression of endogenous *NbenIDA1A* and *IDA*-like genes from citrus (*CitIDA3*) and Arabidopsis (*AtIDA*) in *N. benthamiana* plants. **a** The inoculation of the *CLBV*-based expression construct *clbv3’pr*-NbenIDA1 caused growth cessation and stunting of plants in comparison with plants inoculated with the control vector *clbv3’pr* and with *clbv3’pr*-CitIDA3 and *clbv3’pr*-AtIDA constructs. Close-up of the apical portions of plants inoculated with the control vector *clbv3’pr*, (**b**) and with *clbv3’pro*-NbenIDA1 (**c**), *clbv3’pro*-CitIDA3 (**d**), and *clbv3’pro*-AtIDA (**e**) constructs. (**b** to **e**) show flower development stages of plants inoculated with each of the *CLBV* expression vectors. It is interesting to note that while in (**b**, **d**, and **e**) the series of flower developmental stages ranges from 1 to 5 or 6, in (**c**), only flowers at stages 1, 2 and 7 remain, while flowers at stages 3 to 6 were missing. These flowers are apparently smaller and carry shorter corollas

**Fig. 6:**
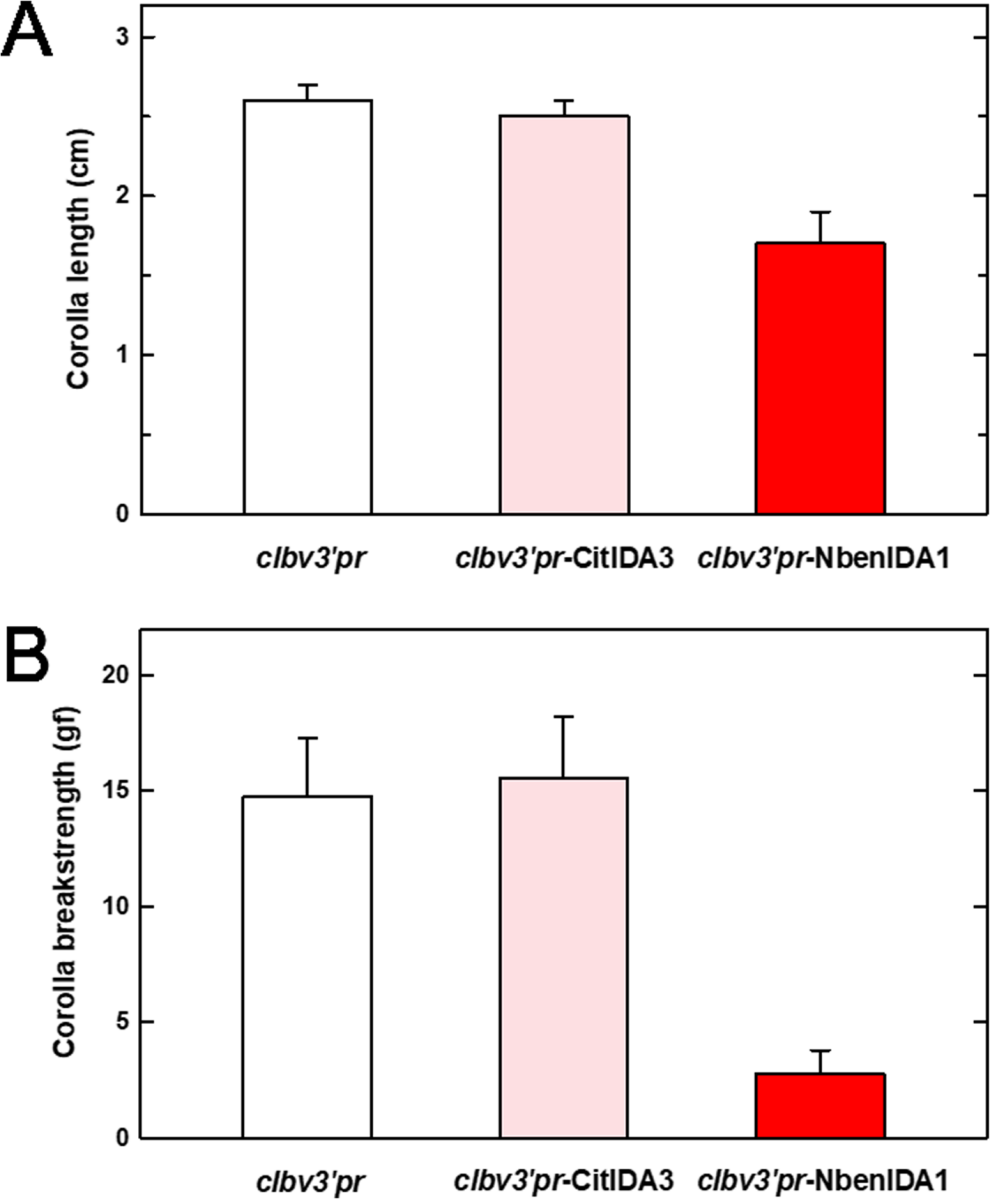
Corolla length and the force required to remove the flower corolla (corolla breakstrength; cBS) in *N. benthamiana* flowers from plants inoculated, respectively, with the control vector (*clbv3’pr*) and constructs expressing the foreign citrus gene *CitIDA3* (*clbv3’pr*-CitIDA3) and the endogenous gene *NbenIDA1A* (*clbv3’pr*-NbenIDA1). **a** Results of the length (cm) of the corolla at flower developmental stage 2. **b** cBS needed to tear out the flower corolla at flower developmental stage 7. Data are the mean of 15 measurements per sample and error bars are standard deviations from the mean

Plants inoculated with *clbv3’pr-*NbenIDA1 vector exhibited three notorious phenotypical changes as related to the other kind of plants (Fig. 5):

i). mature plants showed a dwarf phenotype that affected the whole plant architecture, including leaf area and size, internode and corolla length, flower size and shoot stature (Fig. 5a). As an example of the effect of the over-expression of the endogenous *NbenIDA1A* gene, the length of the flower corollas was measured and compared with those of the plants inoculated with the control vector *clbv3’pr* and with the *clbv3’pr*-CitIDA3 construct (Fig. 5b and c and Fig. 6a). It was evident that the length of the flower corollas corresponding to the plants inoculated with the expression vector *clbv3’pr*-NbenIDA1 was shorter than that of the control plants and also of the plants inoculated with the expression *clbv3’pr*-CitIDA3 construct.

ii). Corollas senesced prematurely, reaching full senescence just after stage 2 of flower development since these plants directly developed necrotic flowers in stage 7 and did not exhibit flowers at intermediate stages (3 to 6) (Fig. 5c).

iii). Corolla abscission was also accelerated (Fig. 6b). Thus, enhanced levels of *NbenIDA1A* resulted in a dramatic decrease of the force required to remove the corollas, reaching cBS values around 3 gf. It should be noted that this effect was the opposite of that observed in *NbenIDA1*-silenced plants (see Fig. 2b).

In contrast to plants inoculated with *clbv3’pr*-NbenIDA1 expression vector, plants inoculated with *clbv3’pr-*CitIDA3 or *clbv3’pr*-AtIDA were basically undistinguishable from those inoculated with the empty *clbv3’pr* vector since their vegetative growth was standard (Fig. 5a) and their flower corollas senesced and detached from the flower receptacles in normal positions (Fig. 5b, d, and e). The evidence that *CLBV* virion constructs systemically spread the infection after inoculation (Additional file 2) suggest that the *IDA*-like genes of Arabidopsis and citrus should be actively expressed throughout the plant.

Therefore, these results showing the phenotype displayed by the over-expression of the endogenous *NbenIDA1A* homeolog add further evidence to the notion that the IDA-HAE signaling module regulates corolla abscission in *N. benthamiana.*

## Discussion

A strategy based on *Citrus leaf blotch virus* (*CLBV*) VIGS vectors was used to characterize the involvement of the pair of *NbenIDA1* homeologs and their potential *NbenHAE* receptors in cell wall dissolution at the base of the corolla tube in *N. benthamiana* flowers. The inoculation of the silencing constructs *clbv3’*-NbenIDA and *clbv3’*-NbenHAE did not produce any obvious effect on plant growth, neither affected the rate of development or size of the major vegetative and reproductive organs (Fig. 1a and c and Additional file 3) but arrested corolla abscission (Fig. 1b and d and Additional file 3). Flowers in plants inoculated with both silencing constructs retained a higher number of corollas attached to the flowers than control plants (Fig. 2a), an observation that may be related to the greater force required to remove corollas from the flower receptacles (Fig. 2b). The arrest of corolla abscission was associated with the preservation of the parenchyma tissue at the base of the corolla tube that, in contrast, was virtually collapsed in normal corollas (Fig. 3). The preservation of the parenchyma tissue at the base of the corolla tube was also reported when the expression of the *BLADE-ON-PETIOLE* gene *NtBOP2* was suppressed in cultivated tobacco plants using an antisense approach [32]. The tobacco gene *NtBOP2* shares similarity to Arabidopsis *BOP2*, which along with its paralog *BOP1*, is involved in the development of floral organ AZs [33, 34]. Thus, the function of *NtBOP2* parallels that of Arabidopsis *BOP1/BOP2* in controlling the formation of the AZ at the base of the corolla tube in flowers of cultivated tobacco plants [32]. The epidermal cell walls at the base of the corolla tube in control *N. benthamiana* flowers remained intact as opposite to the cellular breakdown observed in the parenchyma tissue (Fig. 3) presumably associated with the deposition of lignin. Serafini-Fracassini and co-workers [35] reported that corolla senescence in cultivated tobacco flowers begins at a transition stage between open flower (developmental stage 4) and when the loss of corolla turgidity is evident, a stage that is generally accompanied by the occurrence of a brown ring at the base of the corolla tube (taking place at flower developmental stage 5 in *N. benthamiana*; Additional File 5). At this stage, epidermal cell walls were remarkably auto-fluorescent at the base of the corolla tube [32] suggesting that lignin, one of the most important auto-fluorescent molecules found in plants [36], is deposited in cell walls during senescence. This allow us to speculate with the possibility that the resistance to hydrolysis of the epidermal cell walls at the base of the corolla tube of *N. benthamiana* flowers is related to lignin deposition limiting thus cell wall breakdown to parenchyma tissue. This putative function assigned to lignin deposition maybe linked to recent evidence showing that a lignin-free zone in the pedicel is crucial to seed shattering in rice [37]. It has also been recently reported that the deposition of honeycomb structures of lignin in the walls of cells surrounding Arabidopsis floral organ AZs appears to act as a mechanical brace to specifically localize cell wall dissolution in this tissue [38].

The abscission model proposed by Patterson [39] defined major stages in the abscission pathway, from the differentiation of the AZ to the competence to respond to abscission signals, following by activation of the cell separation process and concluding with the formation of a protective layer on the surface of the separation layer. Two types of AZs have been described: primary AZs and adventitious or secondary AZs [40]. The differentiation of primary AZs occurs simultaneously with the development of lateral organs formed from the shoot apical meristem (SAM). Primary AZ formation takes place only in a limited number of morphological locations on the plant body. By contrast, the differentiation of adventitious AZs begins after the development of lateral organs in a position that is not predetermined by the morphology of the plant. Members of the *BLADE-ON-PETIOLE* (*BOP*) gene family have been implicated in primary AZ formation in several plant species [34, 41] and also in the differentiation of the adventitious AZ at the base of the corolla tube in cultivated tobacco flowers [32]. It is interesting to note that the differentiated primary AZs remain inactive until they acquire the competence to respond to abscission-stimulating signals by triggering the activation of the complex formed between the hormone peptide IDA and its receptors HAE and HSL2 [31, 42–44]. This process of acquiring responsiveness to abscission signals, however, does not appear to occur in adventitious AZs [32, 45]. In primary AZs such as those of Arabidopsis floral organs, *IDA* and *HAESA* gene expression persist in the flower receptacle of the double mutant *bop1 bop2* that is totally deficient in abscission [34]. Thus, primary AZ differentiation and execution of abscission are likely functionally independent processes but success in abscission is dependent on the pre-existence of a functional AZ. The antisense suppression of *NtBOP2* in cultivated tobacco plants [32] displayed the same effect on the anatomical structure of the base of the corolla tube as the suppression of the pair of *NbenIDA1* homeologs and the putative *NbenHAE.1* receptor in *N. benthamiana*, the absence of adventitious AZ formation and the prevention of parenchyma tissue breakdown. Therefore, it can be hypothesized that the differentiation of the adventitious AZ and the execution of abscission at the base of the corolla tube in *Nicotiana* flowers are concurrent and functionally dependent processes since they lead to the same anatomical result, the prevention of adventitious AZ formation and the preservation of the parenchyma tissue.

The *clbv3’*-NbenIDA construct arrested corolla abscission by suppressing the expression of the pair of *NbenIDA1* homeologs while *clbv3’*-NbenHAE produced the same effect on corolla abscission only by suppressing the expression of *NbenHAE.1* (Additional file 4). We selected a silencing trigger sequence to potentially affect both receptor homeologs, *NbenHAE.1* and *NbenHAE.2* (Additional file 2), taking into account that silencing trigger sequences with at least one stretch of more than 21 nucleotides with 100% identity to the target gene sequence may be adequate to induce gene silencing in plants [46, 47]. However, we cannot rule out that the secondary or tertiary structure of the sequence selected to trigger silencing of the pair of *NbenHAE* homeologs might cause problems for the RNA silencing machinery to act specially when base-pairing is imperfect as in *NbenHAE.2*. Regarding the potential function of each of the receptor homeologs, senescence and abscission of floral organs are concurrent processes that occur in flowers after pollination [48]. Both physiological processes involve reactive oxygen species (ROS) contributing to tissue cell death [49, 50], and recent studies in Arabidopsis and tomato implicate IDA-like peptides as potential regulators of ROS homeostasis [51–53]. In Arabidopsis, the mature peptide IDL1 is perceived by HSL2 causing an oxidative burst and programmed cell death (PCD) in the root and then the sloughing off of the root cap cells [51]. The study conducted in tomato suggests that the function of the SlIDA peptide on anther dehiscence depends on the temporal pattern of ROS in the tapetum, although the peptide-receptor complex that triggers the process is not specified [53]. Our study in *N. benthamiana* demonstrates that the receptor NbenHAE.1 is involved in the regulation of corolla abscission, while NbenHAE.2 might be involved in the regulation of corolla senescence triggered by increasing ROS or by other yet undescribed mechanism.

**Fig. 7:**
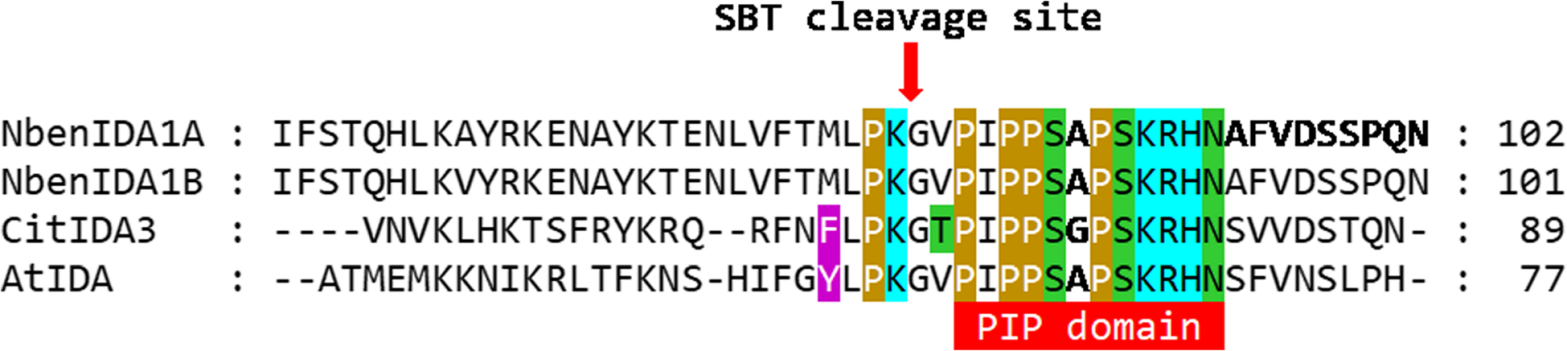
Multiple sequence alignment of the C terminal portion of both *N. benthamiana* NbenIDA1A, citrus CitIDA3 and Arabidopsis AtIDA propeptides. Chemical nature of the amino acid residues upstream of and at the PIP domain using the CINEMA color scheme: polar positive [cyan] and neutral [green] residues; non-polar aliphatic [no color] and aromatic [violet, and goldenrod] residues. SBT, subtilisin-like proteinase

The inoculation of the *clbv3’pr*-NbenIDA1 construct increased the expression of *NbenIDA1A* at the base of the corolla tube of *N. benthamiana* flowers (Fig. 4), negatively affecting the growth of the inoculated plants and the timing of both corolla senescence and abscission (Fig. 5). These disturbances in plant development resemble those produced by the ectopic overexpression of the endogenous *IDA* and the foreign citrus gene *CitIDA3* in Arabidopsis [10, 44]. In contrast, the heterologous ectopic expression of clbv3’pr-CitIDA3 and clbv3’pr-AtIDA expression constructs did not alter the standard phenotype of plants (Figs. 5 and 6). This failure might be associated with a different proteolytic processing machinery required to mature IDA-like propeptides in *N. benthamiana*, since proteolytic cleavage is necessary to produce a functional IDA peptide of optimal length for receptor binding. A plausible candidate for such differences is the subtilase activity. In Arabidopsis, specific subtilases (AtSBT5.2, AtSBT4.12, and AtSBT4.13) have been implicated in the C-terminal processing of the IDA propeptide to yield the mature, active peptide [54]. These subtilases that are involved in cleaving off the two amino acids upstream of the PIP domain, must have active sites suited to bind targets with particular amino acid series. In fact, the five amino acids upstream of the PIP domain are highly similar between IDA and CitIDA3 propeptides (Fig. 7). The amino acids series in both propeptides are constituted by an amino acid with non-polar aromatic side-chain (tyrosine [Y] in IDA and phenylalanine [F] in CitIDA3), an identical core of four amino acids (leucine [L]-proline [P]-lysine [K]-glycine [G]), but an amino acid with non-polar aliphatic side-chain (valine [V] in IDA and threonine [T] in CitIDA3, the later with polar neutral side-chain) (Fig. 7). Thus, the highly similar chemical nature of these two series of amino acids might be related to the fact that heterologous expression of *CitIDA3* in Arabidopsis was effective both in phenocopying the effect of endogenous *IDA* overexpression and in rescuing the abscission deficiency of the *ida2* mutant [10]. In *N. benthamiana*, the amino acids with non-polar aromatic side-chains are substituted in the pair of NbenIDA1 propeptides by methionine (M), an amino acid with non-polar aliphatic side-chain (Fig. 7). Thus, IDA-like propeptides such as those from Arabidopsis and citrus containing amino acids with non-polar aromatic side chains may not successfully bind to active sites of *N. benthamiana* subtilases involved in C-terminal processing. As a result, the enzymatic cleavage of AtIDA and CitIDA3 propeptides is not effective, mature and active peptides are not generated and no phenotype is observed. The feasibility of this scenario will need experimental testing.

## Conclusions

Since the first reports by Jinn and co-workers [42] and Butenko and co-workers [31] showing, respectively, the involvement of the LRR-RLK *HAESA* and the small signaling peptide *IDA* in floral organ abscission, a large body of experimental evidence supports the regulatory role of the signaling module IDA-HAE/HSL2 in organ abscission in Arabidopsis. The identification of a large number of putative Arabidopsis *IDA* orthologs and its *HAESA* family receptors [9] and the abundant experimental results obtained during leaf, flower and fruit abscission in horticultural and fruit crops [for recent review, see 1–3] strongly suggested that the abscission regulatory module IDA-HAE/HSL2 is conserved in angiosperms. However, there is still some reluctance in the scientific community to generalize the function of this module to other plant species [18]. Here, we demonstrate that the pair of *NbenIDA1* homeologs encoding small peptides of the *IDA*-like family and the receptor *NbenHAE.1* control cell wall dissolution in the adventitious AZ formed at the base of the corolla tube and, therefore, the abscission of the corolla in *N. benthamiana* flowers. Besides Arabidopsis, this is the first example claiming that the abscission regulatory module IDA-HAE/HSL2 is conserved in angiosperms.

## Methods

### *CLBV*-based constructs

The CLBV-based vectors used in this survey derived from the infectious *CLBV* clone pBIN35SRbz-CLBV (CLBV-IC) [55], modified by introducing a unique *PmlI* restriction site at the 3’-UTR region to generate the *clbv3’* silencing vector, and introducing a duplicate of the minimum CP sgRNA promoter restoring the *PmlI* restriction [26]. Cloning of *N. benthamiana* silencing trigger gene fragments and coding sequences of *N. benthamiana*, *Citrus clementina*, and Arabidopsis genes (Additional file 2) at the *PmII* restriction sites of the *CLBV*-based vectors was obtained by using standard techniques and appropriate primers (Additional file 5). All constructs were confirmed by sequencing. As a result, we generated constructs to silence the pairs of *NbenIDA1* and *NbenHAE* homeologs named, respectively, *clbv3’*-NbenIDA and *clbv3’*-NbenHAE, and constructs to overexpress the *NbenIDA1A* homeolog and the citrus *CitIDA3* and Arabidopsis *AtIDA* genes, which were named, respectively, *clbv3’pr*-NbenIDA1, *clbv3’pr*-CitIDA3, and *clbv3’pr*-AtIDA (Additional file 2). All constructs were then introduced in *Agrobacterium tumefaciens* cells, strain COR 308 (kindly provided by Dr. C. M. Hamilton, Cornell Research Foundation) using standard protocols.

### Plant growth and inoculations

*N. benthamiana* seeds were obtained from Dr. José Guerri and Dr. Karelia Velázquez of the Centro de Protección Vegetal y Biotecnología (IVIA, Moncada, Spain). Seeds were germinated on nutrient soil and transplanted individually in small pots with an artificial potting mix (50% vermiculite and 50% peat moss) in a plant growth chamber at 20/24 °C (night/day), 60% relative humidity and a 16/8-h light/dark regime.

VIGS constructs were agro-inoculated into *N. benthamiana* leaves as described in [55].

### Corolla breakstrength measurements

The force required to pull the corolla off the flower receptacle (corolla breakstrength, cBS) was measured using a Pesola® spring dynamometer (scale: 100 g; PCE Iberica S. L., Tobarra, Spain). The clamp of the dynamometer was carefully attached to the apical portion of the corolla tube of *N. benthamiana* flowers, and force was applied until the corolla detached from the flower receptacle. Every corolla detachment event was recorded using a video camera. The videos were examined frame-by-frame to determine the magnitude of the force applied to separate the corollas.

### Corolla base anatomy

Flowers from *N. benthamiana* plants inoculated with the empty *clbv3’* vector and *clbv3’*-NbenIDA construct were sampled at developmental stages 5 (onset of corolla senescence with margins of the corolla limb lobes curling inwards) and 6 (corolla limb completely contracted and brown and corolla tube drying).

Samples containing the capsule and the base of the corolla tube attached to the flower receptacle were fixed and embedded in LR White resin (London Resin Co., Woking, Surrey, UK) according to [56]. Longitudinal sections (about 1 μm thick) were cut with a Leica RM2255 microtome (Leica Microsystems, Wetzlar, Germany) using glass knives and fixed to microscope slides. Sections were stained with Toluidine Blue O (CI 52040; Merck, Darmstadt, Germany) after [57] and examined and photographed with a Leica DM LA microscope (Leica Microsystems, Wetzlar, Germany).

### RNA extraction, RT-PCR detection and qPCR analysis

Total RNA was prepared using fresh tissue from both leaves and the base of corolla tubes of *N. benthamiana* flowers, laminar abscission zones of *Citrus clementina* leaves and Arabidopsis flower receptacles using the TRIZOL method [28]. Quality of the isolated total RNA was checked and quantified using the NanoDrop spectrophotometer (Thermo Fisher Scientific, Alcobendas, Spain).

The systemic infection of *N. benthamiana* plants was detected at 18 dpi by conventional RT-PCR using primer pairs KU17L/KU7L flanking the insertion site of the *CLBV* viral vector (Additional file 5). PCR products were visualized by 2% agarose gel electrophoresis and GelRed-staining (Biotium Inc., Hayward, CA, U.S.A.).

Quantitative PCR analysis was performed in three technical replicas using LightCycler® FastStart DNA MasterPLUS SYBR Green I reaction mix and a LightCycler 2.0 instrument (Roche, Basel, Switzerland), using primers for *NbenIDA1A* and *NbenPP2A* listed in Additional file 5. The fluorescence intensity data was obtained through LightCycler Software version 4.1. Three biological replicates for corollas from control and *clbv3’pr*-NbenIDA1 inoculated plants were used. The relative quantification of transcript levels was normalized using the *NbenPP2A* gene [58] against the control and determined using the 2^−ΔΔCt^ method.

## Supplementary Information

**Additional file 1.** Multiple sequence alignments of IDA-like prepropeptides and HAE-like protein kinases. The critical amino acid residues of the IDA-like peptides for interaction in the peptide binding pocket of the HAE-like receptors and the critical and secondary amino acid residues in the peptide binding pocket of the HAE-like receptors are highlighted.

**Additional file 2. **Description of sequences selected to trigger silencing of the pairs of *NbenIDA1* and *NbenHAE* homeologs to generate the constructs of the *CLBV*-based vectors.

**Additional file 3.** Phenotypes of plants showing non-abscissed corollas after inoculation with the silencing constructs clbv3’-NbenIDA and clbv3’-NbenHAE.

**Additional file 4. **Unequivocal identification by RNA sequencing of the silencing of *NbenIDA1* and *NbenHAE* homeologs by constructs *clbv3’*-NbenIDA1 and *clbv3’*-NbenHAE at the base of the corolla tube.

**Additional file 5. **Description of the flower morphology of *Nicotiana benthamiana* and the developmental stages of the life span of the flower corolla.

**Additional file 6.** Primers used in this work.

## Data Availability

All data generated or analysed during this study are included in this published article and its supplementary information files.

## Abbreviations

AZ: Abscission zone
Aux/IAA: Auxin/indole-3-acetic acid
BOP: BLADE-ON-PETIOLE
BP: BREVIPEDICELLUS
cBS: Corolla breakstrength
ERF: Ethylene response factor
GFP: Green fluorescent protein
HAE: HAESA
HSL2: HAESA-like 2
IDA: INFLORESCENCE DEFICIENT IN ABSCISSION
KNAT1: KNOTTED-LIKE FROM ARABIDOPSIS THALIANA 1
PG: Polygalacturonase
PCD: Programmed cell death
ROS: Reactive oxygen species
SAM: Shoot apical meristem
SERK: SOMATIC EMBRYOGENESIS RECEPTOR-LIKE KINASE
TRV: *Tobacco rattle virus*
VIGS: Virus-induced gene silencing
