# Supplementary material for: IDA (INFLORESCENCE DEFICIENT IN ABSCISSION)-like peptides and HAE (HAESA)-like receptors regulate corolla abscission in Nicotiana benthamiana flowers
Source: BMC Plant Biol. 2021 May 21;21:226. doi: 10.1186/s12870-021-02994-8 (PMC8139003; doi:10.1186/s12870-021-02994-8)
Supplement: Supplementary file 1 — Additional file 1. Multiple sequence alignments of IDA-like prepropeptides and HAE-like protein kinases. The critical amino acid residues of the IDA-like peptides for interaction in the peptide binding pocket of the HAE-like receptors and the critical and secondary amino acid residues in the peptide binding pocket of the HAE-like receptors are highlighted. [file 12870_2021_2994_MOESM1_ESM.pdf]

Additional File 1

The small signaling peptides of the IDA-like family of Arabidopsis are synthesized as prepropeptides of 70 to 110 amino acids in length, but must follow an obligatory maturation pathway to become active cell signaling elements (for a recent review, see Stührwohldt et al., 2018). Once proteolytically processed and hydroxylated in the Pro64, the mature IDA peptide can form a complex with the ectodomain of its specific HAE-like receptor kinases HAE and HSL2 through the interaction between specific amino acid residues (Santiago et al., 2016). These are Ser62, Pro64 (hydroxylated), Ser65 and Asn69 in the mature IDA peptide **(Figure 1)**, while the critical amino acid residues for interaction in the peptide binding pocket of HAE receptor are Glu266, Phe289, Ser311, Arg407 and Arg409 **(Figure 2)** (Santiago et al., 2016). In addition to those peptide binding pocket critical amino acid residues in the HAE receptor amino acid sequence there are other secondary residues to ligand-receptor binding (Santiago et al., 2016). These are Ser147, Tyr196, Trp218, Asn240, Asp242, Gln264, Arg288, Asp290, Asn313, Phe315, Lys337, Phe339, Asp361, Ser363, Tyr364 and Ile385.

Estornell LH, Wildhagen M, Pérez-Amador MA, Talón M, Tadeo FR, Butenko MA (2015) The IDA peptide controls abscission in Arabidopsis and Citrus. Front Plant Sci 6 :1–7.

Santiago J, Brandt B, Wildhagen M, Hohmann U, Hothorn LA, Butenko MA, Hothorn M (2016) Mechanistic insight into a peptide hormone signaling complex mediating floral organ abscission. Elife 5: 1-19.

Stührwohldt N, Hohl M, Schardon K, Stintzi A, Schaller A (2018) Post-translational maturation of IDA, a peptide signal controlling floral organ abscission in Arabidopsis. Commun Integr Biol 11: e1395119.

Ventimilla D, Domingo C, González-Ibeas D, Talon M, Tadeo FR (2020) Differential expression of IDA (INFLORESCENCE DEFICIENT IN ABSCISSION)-like genes in Nicotiana benthamiana during corolla abscission, stem growth and water stress. BMC Plant Biol 20: 34.

Wang F, Zheng Z, Yuan Y, Li J, Zhao M (2019) Identification and characterization of HAESA-like genes involved in the fruitlet abscission in litchi. Int J Mol Sci 20: 5945.

Ying P, Li C, Liu X, Xia R, Zhao M, Li J (2016) Identification and molecular characterization of an IDA-like gene from litchi, LcIDL1, whose ectopic expression promotes floral organ abscission in Arabidopsis. Sci Rep 6: 37135.

```
AtIDA     : -----------------MAPCRTMMVLLCFVLFLAASSSCVAAARIG------------- :  30
NsylIDA1  : --MASSSSSSSSSSSSKNKTLYYLICLILAISFLVGYGVEARPGRM----IMEEEEANSR :  54
NtomIDA1  : ----MASSSSSSSSSSKNKTLYYLICLILAISFLLGYGVEARP----------IEEANSR :  46
NtabIDA1A : MASSSSSSSSSSSSSSKNKTLYYLICLILAISFLVGYGVEARPGRM----IMEEEEANSR :  56
NtabIDA1B : ----MASSSSSSSSSSKNKTLYYLICLILAISFLLGYGVEARP----------IEEANSR :  46
NbenIDA1A : -----MASSSSSSSSSKNKTPFYLICLILAISFLVGYGVEARPGRM----IKEEEEANSR :  51
NbenIDA1B : ------MASSSSSSFSKNKTIYYLICLILAISFLLDYGVEARPGRM----IMEGKKANSR :  50
SlycIDA1  : -------MAFSFSSSKTLYLSSKLTCLILVISLLFNYGHIVEASRFGRIMMVEEN---SR :  50
StubIDA4  : -------MAFSFSSSKTLYLSSKLICLILVISLLGGYDHIVEASRFGRMMIMEENQEKSR :  53
CaIDA4    : -------MASSLSSSKSHYFSSKIICLLLVISLLLVG-YGVEASRFGRKMMIEEN--NSR :  50
SmelIDA5  : -------MAPSLSYSKNLYVSKKLICLVLVISLLVGY--GVEGSRFGRMMMGKKEENSRI :  51
AtIDL1    : -----------------MNLSHKTMFMTLYIVFLLIFGSYNATARIG-------PIKLSE :  36
CitIDA3   : -----MASSSSSSSSKCLHISCKQIYLLFLIVIVLIG-SCEAARPGTTMDS--------- :  45
LcIDL1    : ------------MASKAMHLSCKTIFLSCCIILLLIIGSCTATRPGSTMFVEEKPSQLDS :  48

                                           Ser62^AtIDA
                                             Pro64^AtIDA
                                              Ser65^AtIDA
                                                  Asn69^AtIDA

AtIDA     : --ATMEMKKNIKRLTFKNS-HIFGYLPKGVPIPPSAPSKRHNSFVNSLPH- :  77
NsylIDA1  : IFSTQHLKVYRKENAYKTENLLFTMLPKGVPIPPSAPSKRHNAFVDSSPQN : 105
NtomIDA1  : IFSSQHLKVYRKENAYKTENLLFTMLPKGVPIPPSAPSKRHNAVMDSSPQN :  97
NtabIDA1A : IFSTQHLKVYRKENAYKTENLLFTMLPKGVPIPPSAPSKRHNAFVDSSPQN : 107
NtabIDA1B : IFSSQHLKVYRKENAYKTENLLFTMLPKGVPIPPSAPSKRHNAVMDSSPQN :  97
NbenIDA1A : IFSTQHLKAYRKENAYKTENLVFTMLPKGVPIPPSAPSKRHNAFVDSSPQN :  90
NbenIDA1B : IFSTQHLKVYRKENAYKTENLVFTMLPKGVPIPPSAPSKRHNAFVDSSPQN : 101
SlycIDA1  : IFSSQHMKVYKKENAYKVDNLLFTMLPKGIPIPPSAPSKRHNAIEDSTPQN : 101
StubIDA4  : IFSSQHMKEYKKENAYKVDNLLFTMLPKGVPIPPSAPSKRHNAIEDSTPQN : 104
CaIDA4    : LFSSQHMKVYKKENAYKTQNLLFTMLPKGVPIPPSAPSKRHNAIED----- :  96
SmelIDA5  : FSSQVHLKVYKKENSYKIDNLMFTMLPKGIPIPPSGPSKRHNAIEDSTPQN : 102
AtIDL1    : TEIVQTRSRQEIIGGFTFKGRVFHSFSKRVLVPPSGPSMRHNSVVNNLKH- :  86
CitIDA3   : ----VNVKLHKTSFRYKRQ--RFNFLPKGTPIPPSGPSKRHNSVVDSTQN- :  89
LcIDL1    : ETRKTHWRKFETGFQYKGQ--MFSFFPKGTPIPPSGPSKRHNSVVDSTPSD :  97
                                           PIP domain
```

Figure 1. Multiple sequence alignment of selected Arabidopsis and Solanaceae IDA-like prepropeptides possibly involved in organ abscission (Ventimilla et al., 2020) together with the citrus (CitIDA3) and litchi (LcIDL1) prepropeptides replacing the function in abscission of IDA in Arabidopsis (Estornell et al., 2013; Ying et al., 2016). The PIP domain amino acids that directly bind to the ectodomain of the LRR-RLKs HAE/HSL2 are shadowed in green.

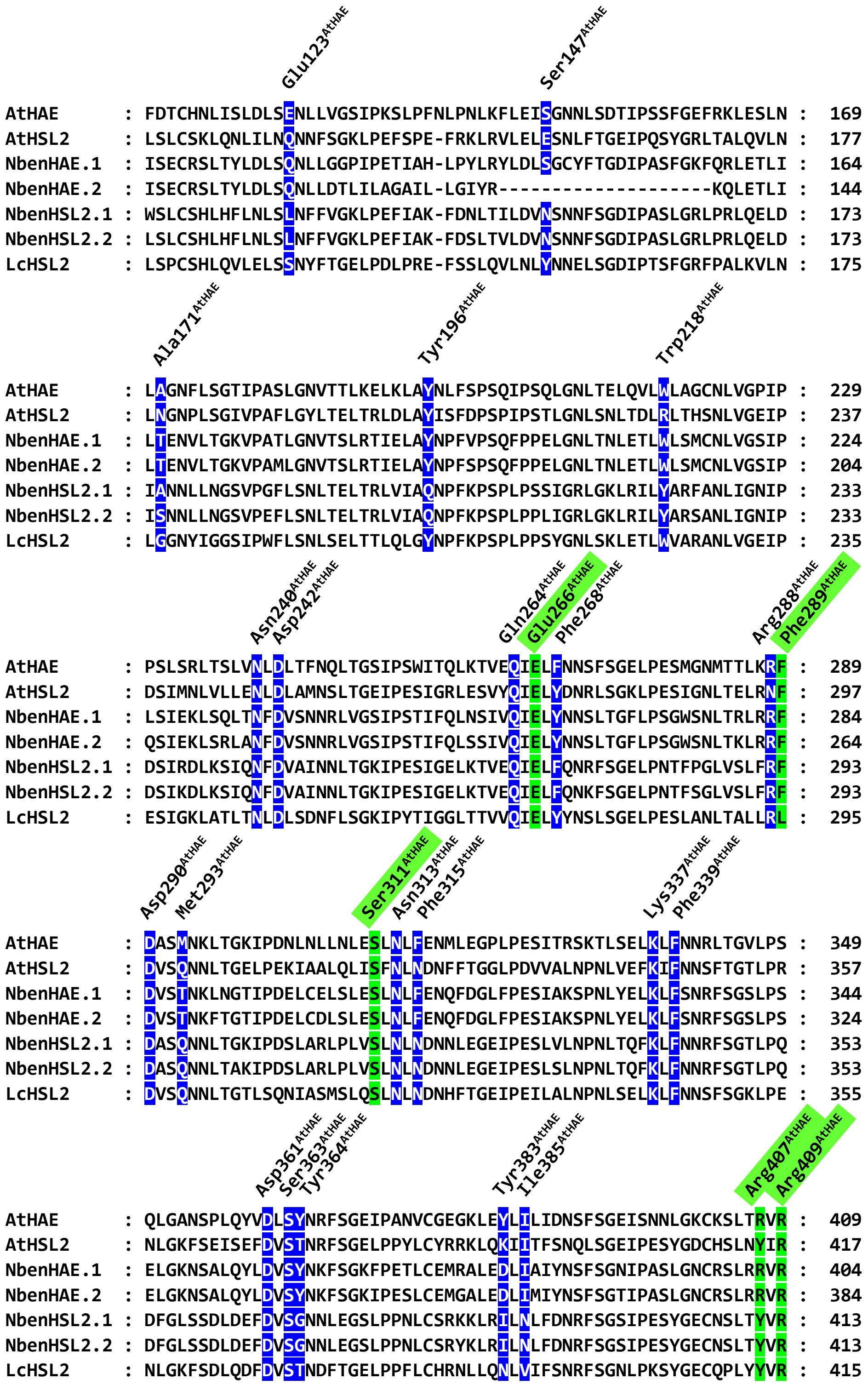

Figure 2. Multiple sequence alignment of selected Arabidopsis and *Nicotiana benthamiana* HAE-like protein kinases (Ventimilla et al., 2020) together with a litchi protein kinase (LcHSL2) replacing the function in abscission of HAE/HSL2 in Arabidopsis (Wang et al., 2019). Critical IDA-interacting amino acid residues in the binding pocket of HAE LRR-RLKs are shadowed in green whereas other amino acid residues also interacting with IDA are shadowed in blue.
