# Supplementary material for: IDA (INFLORESCENCE DEFICIENT IN ABSCISSION)-like peptides and HAE (HAESA)-like receptors regulate corolla abscission in Nicotiana benthamiana flowers
Source: BMC Plant Biol. 2021 May 21;21:226. doi: 10.1186/s12870-021-02994-8 (PMC8139003; doi:10.1186/s12870-021-02994-8)
Supplement: Supplementary file 2 — Additional file 2. Description of sequences selected to trigger silencing of the pairs of NbenIDA1 and NbenHAE homeologs to generate the constructs of the CLBV-based vectors. [file 12870_2021_2994_MOESM2_ESM.pdf]

Additional File 2

To generate the constructs of the *CLBV*-based vectors for the silencing of endogenous *N. benthamiana IDA1* homeologs, a 141-nt linear fragment of *NbenIDA1B* covering a portion of the variable region, the EPIP motif and a portion of the C terminus of the prepropeptide was selected **(Figure 1)**. Similarly, a 191-nt linear fragment of *NbenHAE.1* covering the LRR motifs #11, #12 and #13 of the Leucine-rich repeat domain of the receptor kinase **(Figure 2)** was amplified and used for the silencing of *N. benthamiana HAE* homeologs. It has been experimentally established that silencing trigger sequences with at least one stretch of more than 21 nucleotides with 100% identity to the target gene sequence may be adequate to induce gene silencing in plants (Thomas et al., 2001; Senthil-Kumar et al., 2007). The selected silencing trigger sequence of *NbenIDA1B* had >99% identity with that of *NbenIDA1A* and that of *NbenHAE.1* showed >95% identity with that of *NbenHAE.2*. The two selected silencing trigger sequences did not contain stretches higher than 21 nucleotides with 100% identity with other *IDA*-like peptides **(Figure 1)** and *HAE*-like receptor kinases of *N. bentamiana* **(Figure 3)** suggesting that the two pairs of homeologs (*NbenIDA1* and *NbenHAE*) might be silenced. Both gene fragments were cloned into the *PmlI* restriction site of the *clbv3'* viral vector to obtain the constructs *clbv3'*-NbenIDA1 and *clbv3'*-NbenHAE **(Figure 4)**.

To investigate the effect of the ectopic expression of *IDA*-like genes in *N. benthamiana* plants several other constructs in the *CLBV*-based expression vectors were generated. Regions of *NbenIDA1A* gene from *N. benthamiana*, the *CitIDA3* gene from *Citrus clementina*, and the *IDA* gene from Arabidopsis **(Figure 1)** were cloned into the *clbv3'pr* vector to generate the *clbv3'pr*-NbenIDA1, *clbv3'pr*-CitIDA3 and *clbv3'pr*-AtIDA expression constructs, respectively **(Figure 4)**.

Senthil-Kumar M, Hema R, Anand A, Kang L, Udayakumar M, Mysore KS (2007) A systematic study to determine the extent of gene silencing in *Nicotiana benthamiana* and other Solanaceae species when heterologous gene sequences are used for virus-induced gene silencing. New Phytol 176: 782-791.

Thomas CL, Jones L, Baulcombe DC, Maule AJ (2001) Size constraints for targeting post-transcriptional gene silencing and for RNA-directed methylation in *Nicotiana benthamiana* using a potato virus X vector. Plant J 25: 417-425.

```
NbenIDA1A:  ATGGCTTCCTCCTCCTCTTCCTCTTCTTCTTCTAAAAATAAAACCCCTTTTTACTTAATT  60
NbenIDA1B:  ---ATGGCCTCCTCCTCTTCCTCTTCTTTTTCTAAAAACAAAACCATTTATTATTTAATT  57
NbenIDA2A:  ---------ATGGCTTATTCTACTAATTCTAAAACTTTTTATTTTTCATGGAATTTTATG  51
NbenIDA2B:  ---------ATGGCTTATTCTACTAATTCTAAAACCTTTCATTTTTCATGGAATTTCATT  51
NbenIDA5A:  -----------------ATGATCAGTTTCTTCAGAAGA-AAAGTAGCTCTTATTCTAGTC  42
NbenIDA5B:  -----------------ATGATTAGTTTCTTCAGAAGA-AAAGTACCTCTTATTCTA---  39
NbenIDA4 :  --------------ATGGGGAAAATGAGCTTAAAGACA-ACAATACTAT-----------  34
NbenIDA3A:  --------------ATGTTGAAAAGGTTTAAAAACACA-ACAATATTAGTCTTGCTAC--  43
NbenIDA3B:  --------------ATGTTGAAAAGGTTTAAAAACAAA-ACAATATTAGTGTTGCTGC--  43

NbenIDA1A:  TGTTTGATTCTTGCCATTTC---TTTTCTTGTTGGTTATGGAGTTGAAGCAAGACCAGGA  117
NbenIDA1B:  TGCTTGATTCTTGCCATTTC---TTTTCTTCTTGATTATGGAGTTGAAGCAAGACCAGGG  114
NbenIDA2A:  TGCTTGATTCTTACCCTTTC---TCTTGTTCTTGGCTATGGTGCTGCAGTGAGATCAATG  108
NbenIDA2B:  TGCTTGATTCTTACCCTTTC---TCTTGTTCTTGGCTTTAGTGCTGCAGTGAGATCAATG  108
NbenIDA5A:  TTTTGGATGGCTATTATATTAATCACTATTTTTGGTCATTGTCAT---GGTTCAAGAAGC  99
NbenIDA5B:  ---------GTCTTTATATTAATCACTATTTTTGGTCATTGTCAT---GGTTCAAGAAGC  87
NbenIDA4 :  -------TTGTTGTTTTGCTTCTTTTGATGGTTGACCATGCTTATGCTGCAAGGGCAACG  87
NbenIDA3A:  -TTTCTCTTCATCTTCTTCTGATTTTCTTGGCTGATTATCACCATGCAAATGCAACAAAG  102
NbenIDA3B:  -CATTTCTTCTTATTCTTCTGATATTTATGGCTGATAATTACCATGCAAATGCAACAAAG  102

NbenIDA1A:  AGAATGATAAAGGA---------GGAAGAAGAAGCCAATTCAAGAATATTTTCAACACAA  168
NbenIDA1B:  AGAATGATAATGGA---------GGGAAAAAAAGCAAATTCAAGAATATTTTCAACACAA  165
NbenIDA2A:  GTGGCAACGACGACGA---CGAAGAATAAAGAGGAAGCTTCTGGAATGTTCTCA---GAG  162
NbenIDA2B:  GTGACAACAACAGCGACGACGACGACGAAAGGAGAAGCTTCTGGAATGTTCTCA---GAG  165
NbenIDA5A : AGC------------------------------------TCTCAAGTA---------TTT  114
NbenIDA5B : AGC------------------------------------TCTCAAAAA---------TTT  102
NbenIDA4  : CAC------------------------------------ACACAATTT---------CTC  102
NbenIDA3A : AAC------------------------------------TCACAACTT---------TTT  117
NbenIDA3B : AAC------------------------------------TCACAAGTT---------TTT  117

NbenIDA1A:  CATTTGAAGGCATACAGAAAAGAAAATGCATACAAAACAGAAAATTTGGTATTTACTATG  228
NbenIDA1B:  CATTTGAAGGTATACAGAAAAGAAAATGCATACAAAACAGAAAATTTGGTATTTACTATG  225
NbenIDA2A:  CCTGTGAAAGACTTATACGGTGAAAATGA---ATATTTGAAAGGTGATTGGCTCAATATG  219
NbenIDA2B:  CCCGTGAAAGACTTCTATGGTGAAAA------GAATCTGAAAGAAAATTGGTTCAATATG  219
NbenIDA5A:  AACCCAAG---------TAGCCAAAGAAACTCTCATCGATATGGCCATTTTTGGAACTTA  165
NbenIDA5B:  AACCCGAG---------TAGCCAAAGAAATTCTCATCAATATGGCCATTTTTGGAACTTG  153
NbenIDA4 :  AAAGTTCAGCCTTTGCATATGATG---------AATAAATCTCATCAATTCTCAGAGTCT  153
NbenIDA3A:  AATGTTAAGCCTTTGCCTAATTCTCACAATAATTCTCCTCATACATCATTTTCTCAATCT  177
NbenIDA3B:  AATGTTAAGCCATTGCCTAATTCCCACAATAATTCTCCTCACAGATCATTTTCTCAGTCT  177

NbenIDA1A:  CTACCAAAAGGGGTTCCAATTCCTCCTTCTGCTCCATCTAAGAGGCACAATGCTTTTGTG  288
NbenIDA1B:  CTACCAAAAGGGGTTCCAATTCCTCCTTCTGCTCCATCTAAGAGACACAATGCTTTTGTG  285
NbenIDA2A:  CTACCAAAAGGTGTTCCTATTCCTCCTTCTGCACCATCCAAAAGGCACAATTATTATGTG  279
NbenIDA2B:  CTACCAAAAGGTGTTCCTATTCCTCCTTCTGCACCATCCAAAAGGCACAATTATTATGTG  279
NbenIDA5A:  CTGCCTAAAAGAATTCCAATACCAGCTTCTGGTCCATCAAGAAAACACAATGATATTGGT  225
NbenIDA5B:  TTGCCAAAGAGAATTCCAATACCAGCTTCTGGTCCATCAAGAAAACATAATGATATTGGT  213
NbenIDA4 :  TTGCCAAAAGGGGTCCCAATTCCACCTTCTGCTCCTTCCCAACGGCACAATGGTATCAAC  213
NbenIDA3A:  TTGCCAAAAGGAATCCCTATTCCACCTTCTGCTCCTTCAAAAGGCACAATGGTATCAAC  237
NbenIDA3B:  TTGCCAAAAGGAATACCTGTTCCACCTTCTGCTCCTTCGAAAAGGCACAATGGTATCAAC  237

NbenIDA1A:  GACTCTTCTCCTCAAAATTGA------  309
NbenIDA1B:  GACTCTTCACCTCAAAATTGA------  306
NbenIDA2A:  AACTCTTACCCTTAA------------  294
NbenIDA2B:  AACCCTTACCCTTAA------------  294
NbenIDA5A:  CTTAAGAGTACTTGGAGATTACCCTAA  252
NbenIDA5B:  CTTAAGAGTACTTGGAGATTACCCTGA  240
NbenIDA4 :  CTCAAAAGGCTAATTATTAGGCCTTGA  240
NbenIDA3A:  CTCTAA---------------------  243
NbenIDA3B:  CTCTAA---------------------  243
```

Figure 1. Multiple sequence alignment of all members of the *N benthamiana IDA*-like family. The silencing trigger sequence selected in *NbenIDA1B* is shadowed in red as well as the nucleotides conserved in the rest of the *N. benthamiana IDA*-like family members.

```
ATGCAACTATTCATCTTCTTTTTGAGTAGTCTGCCTTTCATATTTGCTTTAAATCAAGATGGGCTATATCTGCAAAGACTGAAACTT -   87
  M  Q  L  F  I  F  F  L  S  S  L  P  F  I  F  A  L  N  Q  D  G  L  Y  L  Q  R  L  K  L -   29
---------------------------------------------                                       LRR
TCTCTTTCCGACACAGAAGGTGCATTTTCTTCTTGGTCTGAACATGATCTTACCCCCTGTAACTGGACAGGTGTCACCTGTAACGAC -  174
  S  L  S  D  T  E  G  A  F  S  S  W  S  E  H  D  L  T  P  C  N  W  T  G  V  T  C  N  D -   58
motif #1
GCGCCGTCTCCCTCCGTCATCGCCGTTAATCTCTCCGGCGCTTCTCTAGCCGGACCCTTCCCTATATTCCTCTGCCACCTCACTTCA -  261
  A  P  S  P  S  V  I  A  V  N  L  S  G  A  S  L  A  G  P  F  P  I  F  L  C  H  L  T  S -   87

CTTTCATCCCTCTCTCTTTCCAATAATCTTTTAAATTCTAGTCTCCCTCTTTCTATTTCTGAATGTCGTAGCCTCACTTACCTTGAC -  348
  L  S  S  L  S  L  S  N  N  L  L  N  S  S  L  P  L  S  I  S  E  C  R  S  L  T  Y  L  D -  116
          LRR motif #2                                                          LRR
CTTTCTCAGAATCTCCTCGGCGGCCCTATTCCTGAAACAATTGCTCATCTCCCTTACCTCAGATACCTTGATCTTAGCGGGTGCTAT -  435
  L  S  Q  N  L  L  G  G  P  I  P  E  T  I  A  H  L  P  Y  L  R  Y  L  D  L  S  G  C  Y -  145
motif #3                                                            LRR motif #4
TTTACGGGAGATATTCCGGCAAGTTTCGGAAAAATTCCAGCGACTGGAGACTCTTATACTGACTGAAAATGTTCTTACCGGTAAAGTT -  522
  F  T  G  D  I  P  A  S  F  G  K  F  Q  R  L  E  T  L  I  L  T  E  N  V  L  T  G  K  V -  174
                                                       LRR motif #5
CCTGCTACGTTAGGTAATGTAACGAGCCTCAGGACAATTGAACTCGCTTACAACCCATTTGTACCGAGCCAGTTTCCCCCTGAACTT -  609
  P  A  T  L  G  N  V  T  S  L  R  T  I  E  L  A  Y  N  P  F  V  P  S  Q  F  P  P  E  L -  203
                                         LRR motif #6
GGTAACTTGACGAATCTTGAGACATTATGGCTAAGTATGTGTAATCTTGTTGGTTCAATTCCCCTTAGTATTGAGAAATTGAGTCAA -  696
  G  N  L  T  N  L  E  T  L  W  L  S  M  C  N  L  V  G  S  I  P  L  S  I  E  K  L  S  Q -  232
                          LRR motif #7
TTGACTAATTTTGATGTGTCCAATAATAGACTCGTTGGATCGATACCAAGTACAATTTTCCAGCTTAATAGTATTGTCCAAATTGAG -  783
  L  T  N  F  D  V  S  N  N  R  L  V  G  S  I  P  S  T  I  F  Q  L  N  S  I  V  Q  I  E -  261
         LRR motif #8                                                           LRR
CTGTACAATAATTCCCTTACTGGATTTTTGCCTAGTGGATGGTCTAACTTGACGAGATTGAGAAGATTCGATGTGTCGACTAACAAG -  870
  L  Y  N  N  S  L  T  G  F  L  P  S  G  W  S  N  L  T  R  L  R  R  F  D  V  S  T  N  K -  290
motif #9                                                                 LRR motif #10
TTAAATGGGACTATTCCTGATGAGTTGTGTGAATTGTCACTTGAGTCACTCAATTTATTTGAGAATCAATTTGATGGTTTATTTCCA -  957
  L  N  G  T  I  P  D  E  L  C  E  L  S  L  E  S  L  N  L  F  E  N  Q  F  D  G  L  F  P -  319
     LEUCINE-RICH REPEAT DOMAIN                      LRR motif #11
GAAAGTATAGCTAAGTCTCCTAATTTATATGAGCTCAAGTTATTCTCTAACAGATTTTCAGGGTCATTGCCAAGTGAACTAGGCAAG - 1044
  E  S  I  A  K  S  P  N  L  Y  E  L  K  L  F  S  N  R  F  S  G  S  L  P  S  E  L  G  K -  348
                                 LRR motif #12
AACTCAGCTTTACAGTATCTTGACGTTTCATACAACAAATTTTCGGGTAAATTTCCTGAAACTTTGTGTGAAATGCGAGCTTTAGAG - 1131
  N  S  A  L  Q  Y  L  D  V  S  Y  N  K  F  S  G  K  F  P  E  T  L  C  E  M  R  A  L  E -  377
                   LRR motif #13
GATCTTATAGCAATATACAATTCGTTCTCCGGGAATATTCCAGCTAGTCTTGGCAACTGCCGGAGTTTGAGACGTGTCAGGTTTCGG - 1218
  D  L  I  A  I  Y  N  S  F  S  G  N  I  P  A  S  L  G  N  C  R  S  L  R  R  V  R  F  R -  406
LRR motif #14                                                               LRR motif
GGTAATCAGCTATATGGGGAAGTCCCTACTGAGTTTTGGAGTTTGCCTCAGGTTTATCTTTTAGACCTTTTTGGCAATGCATTTTCA - 1305
  G  N  Q  L  Y  G  E  V  P  T  E  F  W  S  L  P  Q  V  Y  L  L  D  L  F  G  N  A  F  S -  435
#15                                                       LRR motif #16
GGGAATATATCACACATGATTTCTGGTGCCAAAAACTTGTCTAACTTACAAATTTCAAGAAACAGAATCTCAGGGGTTATACCTAGT - 1392
  G  N  I  S  H  M  I  S  G  A  K  N  L  S  N  L  Q  I  S  R  N  R  I  S  G  V  I  P  S -  464
                                                 LRR motif #17
GAAATAGGAAAATTGAAGAATTTAGTTGAGTTTTCCGCAAGTCATAATGAGCTAACGGGAGAAATTCCAGGCACACTAGTGCATCTA - 1479
  E  I  G  K  L  K  N  L  V  E  F  S  A  S  H  N  E  L  T  G  E  I  P  G  T  L  V  H  L -  493
                              LRR motif #18
GGTCAGTTAGGAACTCTTGATCTTAGTTTCAATGAGTTATCAGGGGAAATCCCCTTGGGAATTCACACAATGAAGCAAATCAGTGAG - 1566
  G  Q  L  G  T  L  D  L  S  F  N  E  L  S  G  E  I  P  L  G  I  H  T  M  K  Q  I  S  E -  522
            LRR motif #19                                                       LRR
CTTAACTTGGCTAACAATGGGTTTTCGGGGAAAATTCCAGATGAAATTGGGACTTTGCCAGTGCTTAATTATCTTGATCTTTCTGGG - 1653
  L  N  L  A  N  N  G  F  S  G  K  I  P  D  E  I  G  T  L  P  V  L  N  Y  L  D  L  S  G -  551
motif #20                                                                   LRR motif
AATTACTTCTCGGGTGCAATTCCACTCAGCTTGCAAAGCCTGAAGCTTAATAAGCTAAATTTGTCAAGTAATCGGCTGTCGGGGACT - 1740
  N  Y  F  S  G  A  I  P  L  S  L  Q  S  L  K  L  N  K  L  N  L  S  S  N  R  L  S  G  T -  580
#21                                                    LRR motif #22
GTTCCTGCATTTTTTGATAAAGGTGTTTATAGAAATAGCTTTTCAGGAAACCCAAGTTTGTGTCAAGGTGTTGCTGGTCTTTGTACT - 1827
  V  P  A  F  F  D  K  G  V  Y  R  N  S  F  S  G  N  P  S  L  C  Q  G  V  A  G  L  C  T -  609

GCAAAAGGTAGAGGAAAGCGTGAACGATACCTGTGGGCGTTGAGATCTATCTACACAGTTGCTGGCTTTGTTTTTCTTGTCGGGATT - 1914
  A  K  G  R  G  K  R  E  R  Y  L  W  A  L  R  S  I  Y  T  V  A  G  F  V  F  L  V  G  I -  638
   -------------------------                              TRANSMEMBRANE REGION
GCTATGTTCATTTGGAAGTACCAGAAATTCAAGAAAATTAAGAAAGGAATCAGTATTTCAAAGTGGACATCATTCCATAAGCTCGGA - 2001
  A  M  F  I  W  K  Y  Q  K  F  K  K  I  K  K  G  I  S  I  S  K  W  T  S  F  H  K  L  G -  667
              -------------------------------------------------------------------------
TTCAGTGAATTTGAAATACTTTATGGCCTAGATGAAGCTAATGTAATAGGAAATGGAGCTTCAGGAAGAGTTTACAAAGCTGTTCTA - 2088
  F  S  E  F  E  I  L  Y  G  L  D  E  A  N  V  I  G  N  G  A  S  G  R  V  Y  K  A  V  L -  696
-----------------------------
AGCAATGGTGAAGCAGTAGCAGTTAAGAAGCTATGGGAGAGATCAGTTAAAGATGAAACCAGTTTCGGTGCTCTTGAGTCTAATAAA - 2175
  S  N  G  E  A  V  A  V  K  K  L  W  E  R  S  V  K  D  E  T  S  F  G  A  L  E  S  N  K -  725
```

```
GACGAGTTTGAAATGGAAGTTGAAACTCTGGGTAAAATTAGGCACAAGAATATTGTGAGATTGTGGTGCTGTTGTGATACTGGGGGT - 2262
  D  E  F  E  M  E  V  E  T  L  G  K  I  R  H  K  N  I  V  R  L  W  C  C  C  D  T  G  G -  754

AGCAAGCTTTTGGTATATGAGTACATGCCAAATGGAAGTTTGGGTGATTTGCTGCACAGTTGCAATGCCAAATTGTTGGATTGGCCG - 2349
  S  K  L  L  V  Y  E  Y  M  P  N  G  S  L  G  D  L  L  H  S  C  N  A  K  L  L  D  W  P -  783

TTGAGGTTCAAGATAGCTTTGGATGCTGCTGAGGGGCTCTCTTATTTACACCATGATTGTGTTCCTCCAATTGTTCACCGAGATGTT - 2436
  L  R  F  K  I  A  L  D  A  A  E  G  L  S  Y  L  H  H  D  C  V  P  P  I  V  H  R  D  V -  812

AAGTCAAACAACATATTACTGGATGGTGAATTTGGAGCCAAAATATCAGATTTTGGTGTGGCAAAAATTGTTAAAGCAGCCAGCAAA - 2523
  K  S  N  N  I  L  L  D  G  E  F  G  A  K  I  S  D  F  G  V  A  K  I  V  K  A  A  S  K -  841
                                PROTEIN KINASE DOMAIN
GGTGGTGCGGAATCCATGTCTGTGATTGCTGGTTCCTGTGGTTACATTGCACCAGAGTATGCATACACTCTTCATGTGAATGAAAAG - 2610
  G  G  A  E  S  M  S  V  I  A  G  S  C  G  Y  I  A  P  E  Y  A  Y  T  L  H  V  N  E  K -  870

AGCGACATTTATAGCTTTGGAGTGGTCATTTTGGAGCTGGTGACAGGTAAAAGACCAGTTGGTCCAGAGTTTGGGGAGAAAGATCTA - 2697
  S  D  I  Y  S  F  G  V  V  I  L  E  L  V  T  G  K  R  P  V  G  P  E  F  G  E  K  D  L -  899

GCTACTTGGGTACGCACCACCTTGAACGAGAAAGGAGTTGATCAGTTGCTCGACCCAAATTTGAATTCCAACTTCAAAGAACATATA - 2784
  A  T  W  V  R  T  T  L  N  E  K  G  V  D  Q  L  L  D  P  N  L  N  S  N  F  K  E  H  I -  928

TGCAAGCTTCTTGATATTGGTCTATGTTGTCTTAACCACATTCCAGCTAATCGCCCCTCAATGCGCAGAGTGGTGAAAATGCTCCAA - 2871
  C  K  L  L  D  I  G  L  C  C  L  N  H  I  P  A  N  R  P  S  M  R  R  V  V  K  M  L  Q -  957

GAATCAGTTCCTTACAATGTGCCAGGGATGGTAAACAAGAATGGTAAACTTCTCCCTTACTTTTTTCCGAAATCAGTCTAG       - 2952
  E  S  V  P  Y  N  V  P  G  M  V  N  K  N  G  K  L  L  P  Y  F  F  P  K  S  V  *       -  983
```

Figure 2. Nucleotide and deduced amino acid sequence of *NbenHAE.1*. Functional domains and motifs were annotated using InterProScan 5 (www.ebi.ac.uk/interpro/search/sequence/) and LRRsearch (www.lrrsearch.com). The sequence of the silencing trigger sequence is shadowed in red, the LRR motifs in dark blue, the transmembrane region in green and the protein kinase in yellow.

```
NbenHAE.1 :  CCTAGTGGATGGTCTAACTTGACGAGATTGAGAAGATTCGATGTGTCGACTAACAAGTTA  873
NbenHAE.2 :  CCTAGTGGATGGTCTAACCTGACAAAATTGAGAAGATTCGATGTCTCAACTAACAAGTTT  813
NbenHSL2.1:  AACACG---TTTCCGGGACTTGTTTCTCTGTTCAGGTTTGACGCTTCTCAGAACAATCTC  891
NbenHSL2.2:  AACACG---TTTTCGGGACTTGTTTCTCTGTTCAGGTTTGATGCTTCTCAGAACAATCTC  891
NbenHSL1.1:  GTGAATGGGTGGTCGAAAATGACGGCGTTAAGGCGACTCGACGTGTCCATGAATCGGGTC  876
NbenHSL1.2:  GTGAGTGGGTGGTCGAAAATGACGGCGTTAAGGCGACTCGACGTGTCGATGAATCGGGTC  876

NbenHAE.1 :  AATGGGACTATTCCTGATGAGTTGTGTGAATTGTCACTTGAGTCACTCAATTTATTTGAG  933
NbenHAE.2 :  ACTGGTACTATTCCTGATGAGTTGTGTGATTTGTCACTTGAGTCACTCAACTTATTTGAG  873
NbenHSL2.1:  ACGGGAAAAATACCTGATAGCCTTGCCCGTTTGCCCTTAGTATCTTTGAATCTCAATGAT  951
NbenHSL2.2:  ACGGCAAAAATACCTGATAGCCTTGCCCGTTTGCCGCTAGTATCTTTGAATCTCAATGAT  951
NbenHSL1.1:  ACGGGTACGGTTCCTAGGGAGTTGTGTGAGTTGCCACTCGAGTCGCTGAATCTTTATGAG  936
NbenHSL1.2:  A-----------------------------------------------------------  877

NbenHAE.1 :  AATCAATTTGATGGTTTATTTCCAGAAAGTATAGCTAAGTCTCCTAATTTATATGAGCTC  993
NbenHAE.2 :  AATCAATTTGATGGTTTATTTCCAGAAAGTATAGCTAAGTCTCCTAATTTGTATGAGCTC  933
NbenHSL2.1:  AACAATTTAGAAGGCGAAATTCCAGAAAGTTTAGTTCTTAACCCGAATCTTACTCAGTTC  1011
NbenHSL2.2:  AACAATTTAGAAGGCGAAATTCCAGAAAGTTTATCTCTAAACCCGAATCTTACTCAGTTC  1011
NbenHSL1.1:  AACCAAATGTTCGGTGAATTGCCACAAGGCATTGCGAATTCGCCGAATTTGTATGAGTTG  996
NbenHSL1.2:  ------------------------------------------------------------  877

NbenHAE.1 :  AAGTTATTCTCTAACAGATTTTCAGGGTCATTGCCAAGTGAACTAGGCAAGAACTCAGCT  1053
NbenHAE.2 :  AAGTTATTCTCTAACAGATTTTCAGGGTCACTGCCTAGTGAACTAGGAAAGAACTCAGCT  993
NbenHSL2.1:  AAGCTCTTTAACAACAGATTTTCTGGTACTTTACCTCAAGATTTTGGTTTAAGTTCTGAT  1071
NbenHSL2.2:  AAGCTCTTTAACAACAGATTTTCAGGTACTTTACCTCAAGATTTTGGTTTAAGTTCAGAT  1071
NbenHSL1.1:  CGGCTCTTTCACAACCGTTTCAATGGGAGTTTACCTAATGATCTTGGGAAGAATTCGCCT  1056
NbenHSL1.2:  ------------------------------------------------------------  877

NbenHAE.1 :  TTACAGTATCTTGACGTTTCATACAACAAATTTTCGGGTAAATTTCCTGAAACTTTGTGT  1113
NbenHAE.2 :  TTACAGTATCTTGATGTTTCATACAACAAATTTTCTGGTAAAATTCCAGAAAGTCTGTGT  1053
NbenHSL2.1:  TTGGATGAGTTTGATGTCTCTGGCAATAATCTTGAAGGTTCTTTGCCGCCCAACTTATGT  1131
NbenHSL2.2:  TTGGATGAGTTTGATGTCTCTGGCAATAATCTTGAAGGTTCTTTGCCGCCCAACTTATGT  1131
NbenHSL1.1:  TTGTTGTGGATTGATGTGTCTGAGAATAAATTTTCGGTGAAATTCCGGAAAATTTATGT  1116
NbenHSL1.2:  ------------------------------------------------------------  877
```

Figure 3. Multiple sequence alignment of all members of the *N benthamiana HAE*-like family. The silencing trigger sequence selected in *NbenHAE.1* is shadowed in red as well as the nucleotides conserved in the rest of the *N. benthamiana HAE*-like family members.

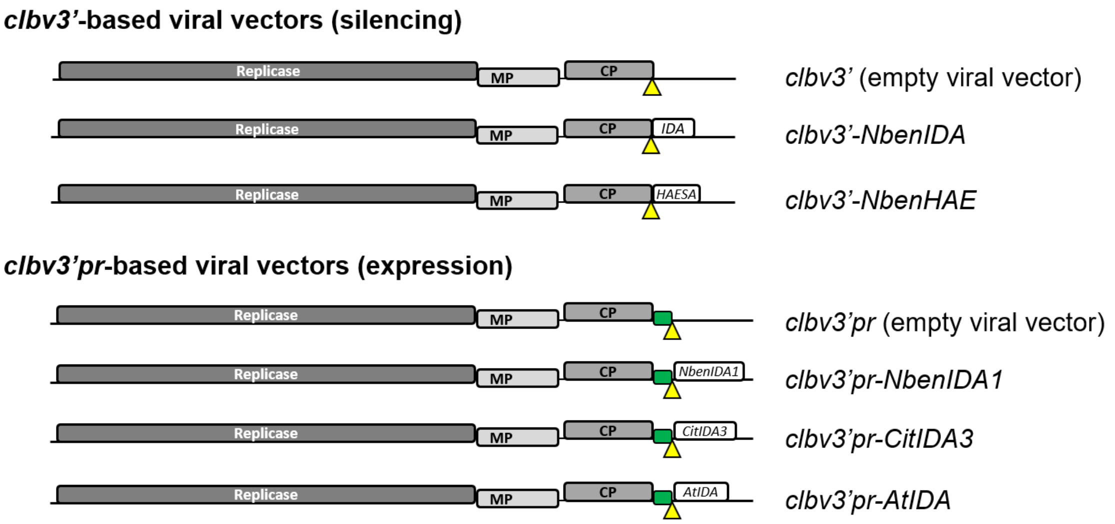

Figure 4. *CLBV*-based viral vectors and constructs used in the study. Green boxes and yellow triangles represent, respectively, the duplicated CP sgRNA promoter and the *PmlI* restriction site added for cloning.

Systemic spread of the infection was assessed by RT-PCR detection of the *CLBV* virions constructs in non-inoculated upper leaves of *N. benthamiana* plants, 20 days post inoculation. *CLBV* was detected in all plants infected with all constructs showed in Figure D, indicating that the modified *CLBV* virions retained their capacity to systemically infect *N. benthamiana* plants **(Figure 5)**.

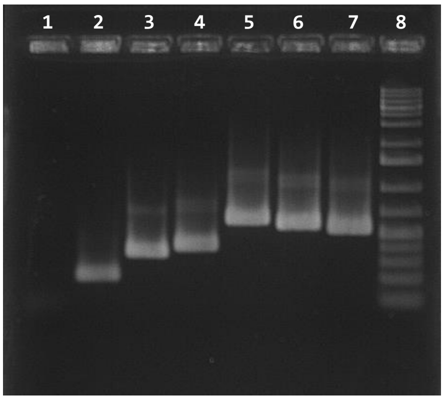

Figure 5. Reverse-transcriptase polymerase chain reaction analysis of total RNA from upper leaves of agro-inoculated *N. benthamiana* plants 20 days post inoculation. Line 1: healthy, non-inoculated plant; line 2: *clbv3'*-control; line 3: *clbv3'*-NbenIDA; line 4: *clbv3'*-NbenHAE; line 5: *clbv3'pr*-NbenIDA1; line 6: *clbv3'pr*-CitIDA3; line 7: *clbv3'pr*-AtIDA; line 8: 1-Kb Plus molecular size ladder.
