# Supplementary material for: IDA (INFLORESCENCE DEFICIENT IN ABSCISSION)-like peptides and HAE (HAESA)-like receptors regulate corolla abscission in Nicotiana benthamiana flowers
Source: BMC Plant Biol. 2021 May 21;21:226. doi: 10.1186/s12870-021-02994-8 (PMC8139003; doi:10.1186/s12870-021-02994-8)
Supplement: Supplementary file 3 — Additional file 3. Phenotypes of plants showing non-abscissed corollas after inoculation with the silencing constructs clbv3’-NbenIDA and clbv3’-NbenHAE. [file 12870_2021_2994_MOESM3_ESM.pdf]

Additional File 3

Abscission phenotype of *Nicotiana benthamiana* plants inoculated with viral constructs

Clbv3' **(control)** | ***Clbv3'*****-NbenIDA** | ***Clbv3'*****-NbenHAE**

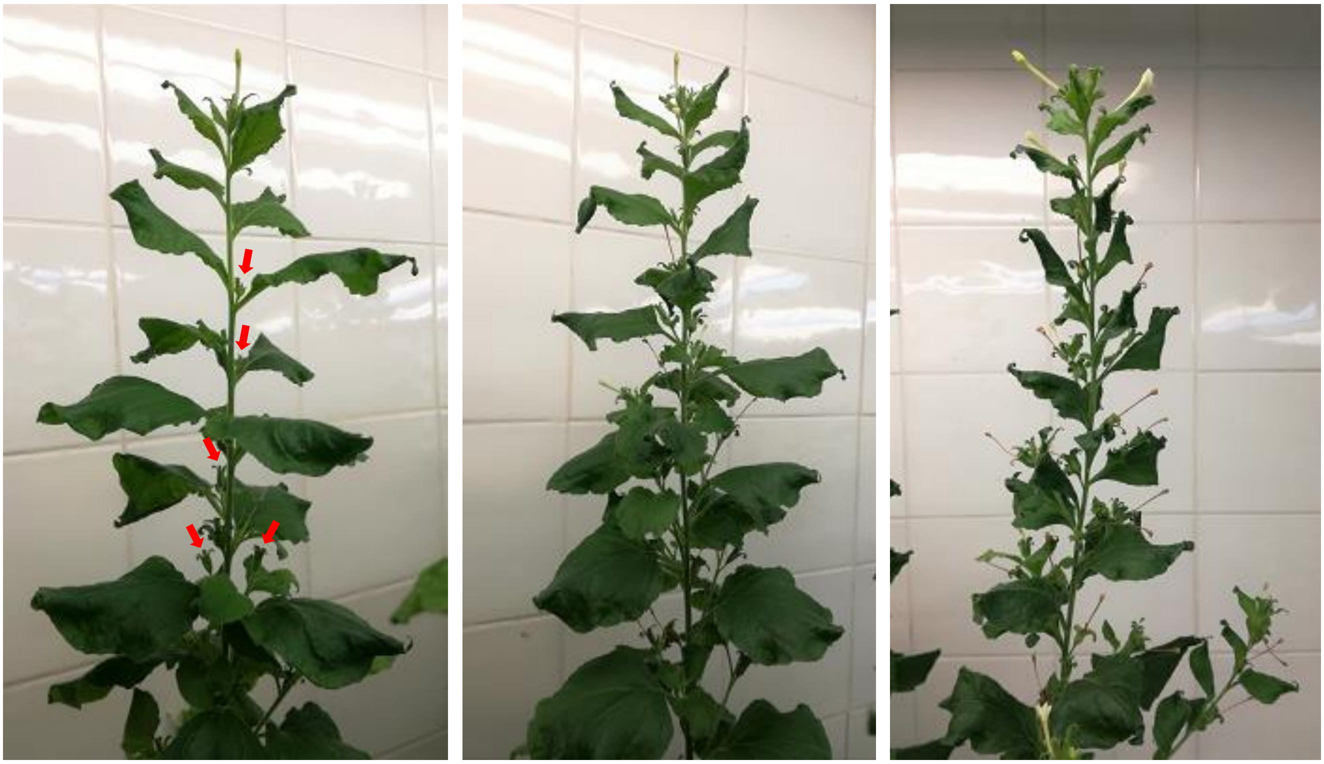

The red arrows in the image showing a plant inoculated with the *clbv3'* vector (control) highlight to flowers that have already shed their corollas. In contrast, inoculation of the silencing constructs *clbv3'*-NbenIDA and *clbv3'*-NbenHAE causes the arrest of corolla abscission.
