# Supplementary material for: IDA (INFLORESCENCE DEFICIENT IN ABSCISSION)-like peptides and HAE (HAESA)-like receptors regulate corolla abscission in Nicotiana benthamiana flowers
Source: BMC Plant Biol. 2021 May 21;21:226. doi: 10.1186/s12870-021-02994-8 (PMC8139003; doi:10.1186/s12870-021-02994-8)
Supplement: Supplementary file 4 — Additional file 4. Unequivocal identification by RNA sequencing of the silencing of NbenIDA1 and NbenHAE homeologs by constructs clbv3’-NbenIDA1 and clbv3’-NbenHAE at the base of the corolla tube. [file 12870_2021_2994_MOESM4_ESM.pdf]

Additional File 4

An RNA sequencing (RNA-seq) approach was used to unequivocally reveal the presence of the targets of *Nicotiana benthamiana IDA*-like and *HAE*-like gene families involved in the prevention of corolla abscission by hindering parenchyma tissue breakdown at the base of corolla tubes. The RNA-seq method used for gene target identification is described in detail at the end of this Additional File.

Three cDNA libraries of *N. benthamina* corolla bases from flowers at developmental stage 4 collected from plants inoculated, respectively, with the empty *clbv3'* vector (CLBV) and the silencing constructs *clbv3'*-NibenIDA (IDAsil) and *clbv3'*-NibenHAE (HAEsil) were prepared for Illumina Paired-End sequencing. After trimming and removing the adaptors, contamination and low-quality sequences, the Illumina sequencing generated 73,524,436 good reads that were mapped to the transcriptome and genome sequences of *N. benthamiana* (Bombarely et al. 2012). The Integrated Genome Viewer (IGV; Robinson et al., 2011; Thorvaldsdóttir et al., 2013) was used for visualization of short-read sequence alignments in single diagrams called Sashimi plots (Katz et al., 2015).

Since coding sequences (CDS) of *NbenIDA1A* and *NbenIDA1B* homeologs exhibited >92% nucleotide identity and the selected silencing trigger sequence of *NbenIDA1B* had >99% identity with that of NbenIDA1A **(Additional File 2)**, it could be predicted that both homeologs might be silenced by *clbv3'*-NbenIDA VIGS construct. The BAM files for the three libraries mapped reads from the genomic regions of the *NbenIDA1* pair of homeologs **(Figure 1)**. Reads of the CLBV and HAEsil libraries mapped the complete predicted sequence, without introns, of the *NbenIDA1* pair of homeologs. However, reads in the IDAsil library only mapped a fragment of the sequence of both homeologs that matched the silencing trigger sequence of the *clbv3'*-NbenIDA construct. The coverage range for the IDAsil library was very high (>3000 and >1000, respectively, for each homeolog) suggesting that the silencing construct was very active in the corolla base at flower developmental stage 4. Therefore, the silencing construct *clbv3'*-NbenIDA appeared to be very efficient producing a strong knock-down effect on the expression of both *NbenIDA1A* and *NbenIDA1B* homeologs.

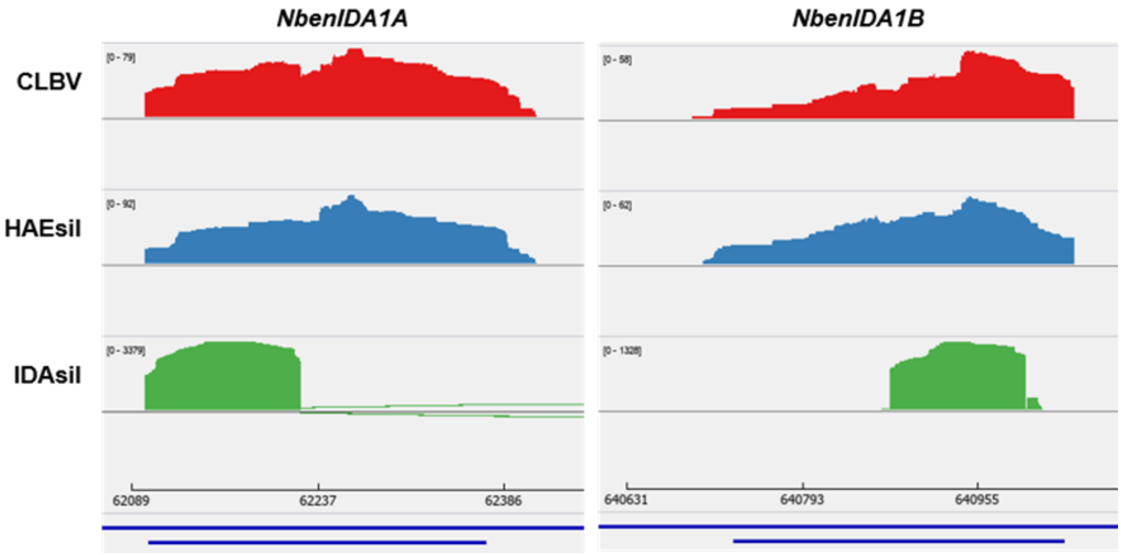

Figure 1. View of *NbenIDA1* pair of homeologs for the Integrated Genome Viewer (IGV). IGV-Sashimi plots showing the read coverage and transcript structures. The histograms represent the sum of the aligned sequencing reads along the genome. Red histograms represent coverage for the CLBV cDNA library (*clbv3'* control vector), blue for the HAEsil cDNA library (*clbv3'*-NbenHAE silencing construct) and green for the IDAsil cDNA library (*clbv3'*-NbenIDA silencing construct). Numbers in square brackets indicate coverage range. Below the histograms is shown in blue the transcript structure of each gene experimentally verified in this study.

The fragment of *NbenHAE.1* selected as silencing trigger sequence showed >95% identity with that of *NbenHAE.2* and contained stretches higher than 21 nucleotides with 100% identity at least in the first half of the sequence **(Additional File 2)**. Therefore, it would be possible that both *NbenHAE* homeologs might be silenced. Reads of the CLBV and IDAEsil libraries mapped the complete predicted sequence of *NbenHAE1* but those in the HAEsil library only mapped a fragment of the sequence that matched the silencing trigger sequence of the *clbv3'*-NbenHAE construct **(Figure 2)**. The coverage range for the HAEsil library was very high (almost 27000) suggesting that as in the case of the other silencing construct, *clbv3'*-NbenHAE appeared to be very active for a strong knock-down effect on the expression of *NbenHAE.1* in the corolla base at flower developmental stage 4. However, reads of all three libreries mapped the complete predicted sequence of *NbenHAE.2* even though the silencing triggered sequence showed >95% identity **(Additional File 2)**. Therefore, the silencing construct *clbv3'*-NbenHAE appeared to be very efficient producing only a strong knock-down effect on the expression of *NbenHAE.1* but no effect on *NbenHAE.2*.

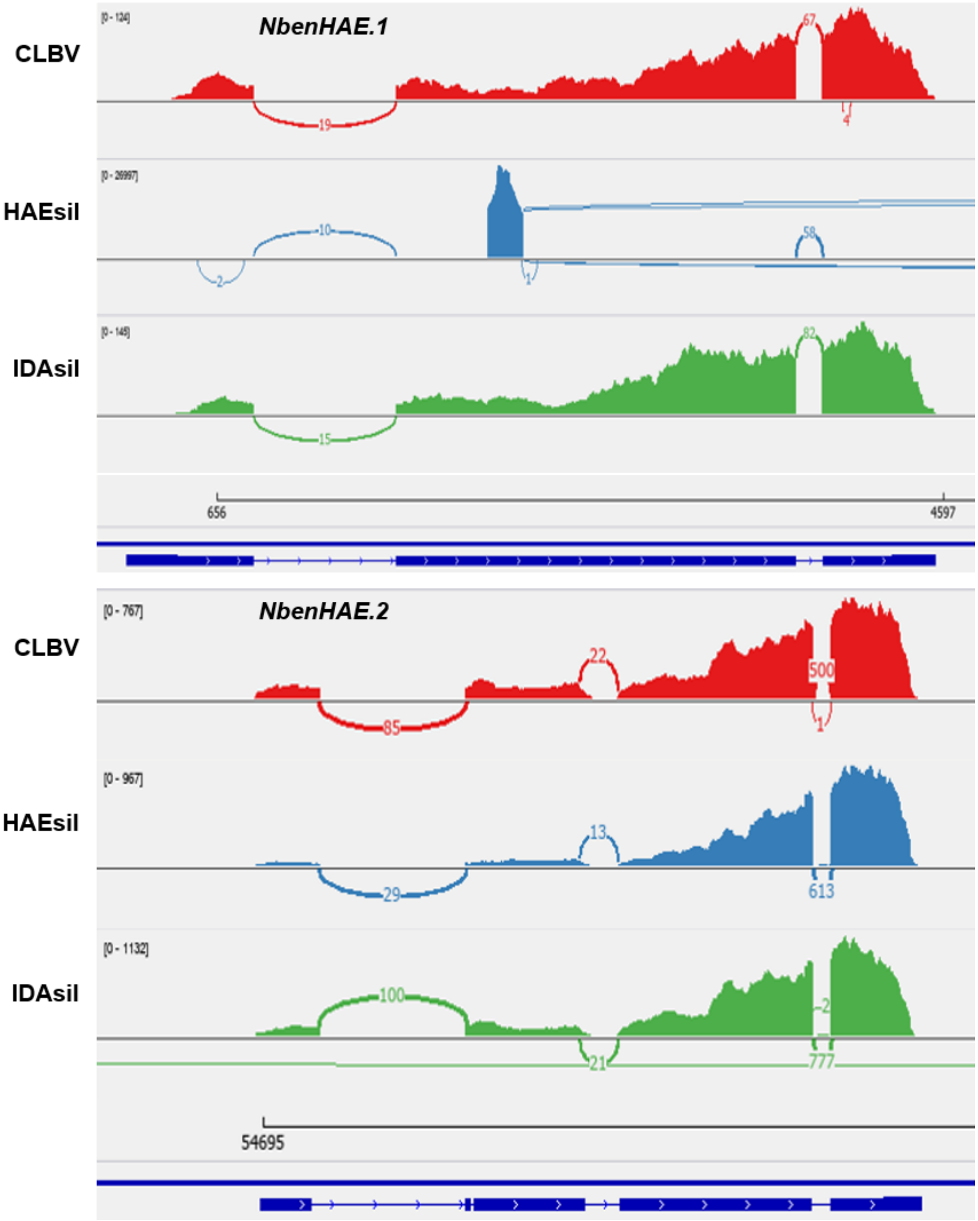

Figure 5.11. View of *NbenHAE* pair of homeologs for the Integrated Genome Viewer (IGV). IGV-Sashimi plots showing the read coverage and transcript structures. The histograms represent the sum of the aligned sequencing reads along the genome. Red histograms represent coverage for the CLBV cDNA library (*clbv3'* control vector), blue for the HAEsil cDNA library (*clbv3'*-NbenHAE silencing construct) and green for the IDAsil cDNA library (*clbv3'*-NbenIDA silencing construct). Numbers in square brackets indicate coverage range. Junction reads are plotted as arcs, and the number of reads aligned to the junction spanning the exons connected by the arc is indicated. Below the histograms is shown in blue the transcript structure of each gene experimentally verified in this study.

RNA-sequencing method

Total RNA from the base of corolla tubes in flowers from plants inoculated with the empty *clbv3'* vector and the silencing constructs *clbv3'*-NibenIDA and *clbv3'*-NibenHAE was isolated using acid phenol extraction and lithium chloride precipitation method as described in (Ecker and Davis, 1987). Quality of the isolated total RNA was checked and quantified using the NanoDrop (Thermo Fisher Scientific, Alcobendas, Madrid).

The isolated total RNA was used for library construction. Pair-end Libraries were prepared using the TruSeq™ RNA sample preparation kit (Illumina Inc.,) according to manufacturer's protocol. Briefly, 0.5 μg of total RNA was used for poly-A based mRNA enrichment selection using oligo-dT magnetic beads followed by fragmentation by divalent cations at elevated temperature resulting into fragments of 80-250 nt, with the major peak at 130 nt. First strand cDNA synthesis by random hexamers and reverse transcriptase was followed by the second strand cDNA synthesis performed using RNAseH and DNA Pol I. Double stranded cDNA was end repaired, 3'adenylated and the 3′- "T" nucleotide at the Illumina adaptor was used for the adaptor ligation. The ligation product was amplified with 15 cycles of PCR.

Each pair-end library was sequenced using TruSeq SBS Kit v3-HS, in paired end mode with the read length 2x76bp. A minimum of 50 million paired end reads for each sample were generated on HiSeq2000 (Illumina, Inc) following the manufacturer's protocol. Images analysis, base calling and quality scoring of the run were processed using the manufacturer's software Real Time Analysis (RTA 1.13.48) and followed by generation of FASTQ sequence files by CASSAVA. FASTQ files were pre-processed with Trimmomatic 0.38 (Bolger et al., 2014), and reads with average quality smaller than 25 and shorter than 36 bases were filtered.

The transcriptome and the genome sequences of *N. benthamiana* (Bombarely et al., 2012) were used as reference for sequence read mapping using the STAR RNA-seq aligner with default parameters (Dobien et al., 2013) as implemented in the OmicsBox suite (https://www.biobam.com/omicsbox). The mapping of short sequencing reads (FASTQ) was performed using RSEM software package, which allocates multi-mapping reads among transcripts using an expectation maximization approach (Li et al., 2011), based on a fast gapped-read alignment with Bowtie 2 (Langmead and Salzberg, 2012).

Bolger AM, Lohse M, Usadel B (2014) Trimmomatic: a flexible trimmer for Illumina sequence data. Bioinformatics 30:2114-2120.

Bombarely A, Rosli HG, Vrebalov J, Moffett P, Mueller LA, Martin GB (2012) A Draft Genome Sequence of Nicotiana benthamiana to Enhance Molecular Plant-Microbe Biology Research. Mol Plant-Microbe Interact 25: 1523-1530.

Dobin A, Davis CA, Schlesinger F, Drenkow J, Zaleski C, Jha S, Batut P, Chaisson M, Gingeras TR (2013) STAR: ultrafast universal RNA-seq aligner. Bioinformatics 29:15-21.

Ecker JR, Davis RW (1987) Plant defense genes are regulated by ethylene. Proc Natl Acad Sci USA 84:5202-5206.

Langmead B, Salzberg SL (2012) Fast gapped-read alignment with Bowtie 2. Nat Methods 9:357-359.

Li B, Dewey CN (2011) RSEM: accurate transcript quantification from RNA-Seq data with or without a reference genome. BMC Bioinformatics 12:323.

Robinson JT, Thorvaldsdóttir H, Winckler W, Guttman M, Lander ES, Getz G, Mesirov JP (2011) Integrative genomics viewer. Nat Biotechnol 29: 24-26.

Thorvaldsdóttir H, Robinson JT, Mesirov, JP (2013) Integrative Genomics Viewer (IGV): high-performance genomics data visualization and exploration. Brief Bioinf 14: 178-192.
