# Supplementary material for: IDA (INFLORESCENCE DEFICIENT IN ABSCISSION)-like peptides and HAE (HAESA)-like receptors regulate corolla abscission in Nicotiana benthamiana flowers
Source: BMC Plant Biol. 2021 May 21;21:226. doi: 10.1186/s12870-021-02994-8 (PMC8139003; doi:10.1186/s12870-021-02994-8)
Supplement: Supplementary file 5 — Additional file 5. Description of the flower morphology of Nicotiana benthamiana and the developmental stages of the life span of the flower corolla. [file 12870_2021_2994_MOESM5_ESM.pdf]

Additional File 5

Nicotiana benthamiana plants develop solitary flowers that arise from the leaf axils and also from the main and secondary stem internodes. They are pentamerous, where the perianth is composed of a calyx of five sepals, and five fused petals forming a sympetalous tubular corolla capped by a five-lobed limb **(Figures 1 and 2B)**. The five stamens are epipetalous and the gynoecium is bicarpellate and formed by a short bilocular ovary with central placentation (Fourquin and Ferrándiz, 2012). From the apical portion of the ovary starts an elongated style capped by a round flat stigma which is inserted into the upper half of the corolla tube. The internal floral structures show homostylity with styles of uniform length and equal to that of the stamens.

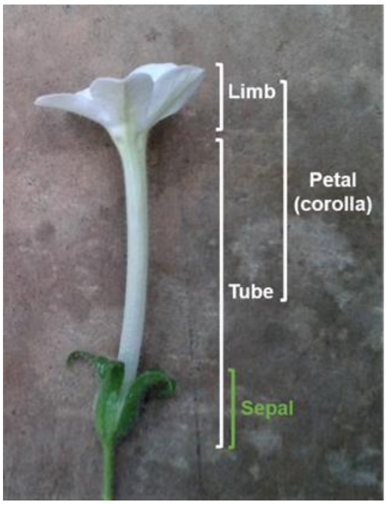

Figure 1. External parts of the flower of *Nicotiana benthamiana*.

Table 1. Morphological markers of the flower development of *N. benthamiana*.

Developmental stage	Morphological markers
1	Corolla tube bulge above calyx; corolla limb tips closed
2	Corolla tube bulge fully elongated, enlarging horizontally and cup-shaped; corolla limb tips closed; anther tapetum degenerated and connective tissue separating the pollen sacs starting to degrade[a]
3	Onset of anthesis; corolla limb halfway open; corolla limb lobes expanding horizontally; bilocular anthers with pollen grains filling the locules[a]
4	Whole open flower; corolla limb lobes fully expanded; sharp pinwhite border at top of tube cup-bulge; anthers dehisce along the stomium
5	Onset of corolla senescence; margins of the corolla limb lobes curl inwards; corolla tube shows an appreciable loss of turgidity
6	Corolla limb completely contracted and brown; corolla tube is drying
7	Corolla death; corolla tube is almost completely dried and brown; corolla tube can be easily detached by its base

[a] Anatomical parameters of anther development taken from reported *N. tabacum* data (Koltunow et al., 1990)

The life span of the corolla can be divided into seven stages associated with the development of the *N. benthamiana* flower **(Table 1)**. These stages include corolla tube elongation, corolla opening, and corolla collapse and senescence **(Figure 2A)**. The corolla tube is fully elongated between flower developmental stages 1 and 2 and flower anthesis extends between stages 3 and 4. It is also between stages 3 and 4 of flower development that anther dehiscence occurs **(Figure 2B)**. The senescence of the corolla begins in the flower developmental stage 5 and extends until stage 7. The senescence process is characterized by a gradual loss of corolla tube turgidity and the emergence of a noticeable brown ring at the base of the corolla tube **(Figure 2C)**. This brown basal ring marks a band of apparently low mechanical resistance, a potential abscission zone through which the corolla will detach from the flower receptacle. The fruits in the Solanaceae family are predominantly capsules and berries, with dry capsules in the subfamily Nicotianoideae (Knapp, 2002). The enlargement of the capsule also contributes to the disintegration of the base of the corolla tube and their shedding of the flower receptacle. Thus, unlike the free-petaled choripetalous corollas of, for example, Arabidopsis in which the petals are separated by their attachment point to the receptacle (McKim et al., 2008), in the sympetalous corollas of *Nicotiana* flowers the cell separation process seems to occur at the base of the corolla tube (Wu et al., 2012; **Figure 2C**). The flower corolla can be easily separated in flower stage 7 by a gentle pulling out. In later stages, structural damage to the base of the corolla tube is enhanced by capsule development until the corolla base is completely disintegrated and the senescent corolla is only weakly held by the pointed apical end of the capsule.

Fourquin C, Ferrándiz C (2012) Functional analyses of *AGAMOUS* family members in *Nicotiana benthamiana* clarify the evolution of early and late roles of C-function genes in eudicots. Plant J 71: 990-1001.

Knapp S (2002) Tobacco to tomatoes: a phylogenetic perspective on fruit diversity in the Solanaceae. J Exp Bot 53: 2001-2022.

Koltunow AM, Truettner J, Cox KH, Wallroth M, Goldberg RB (1990) Different Temporal and Spatial Gene Expression Patterns Occur during Anther Development. Plant Cell 2: 1201-1224.

McKim SM, Stenvik G-E, Butenko M a, Kristiansen W, Cho SK, Hepworth SR, Aalen RB, Haughn GW (2008) The BLADE-ON-PETIOLE genes are essential for abscission zone formation in Arabidopsis. Development 135: 1537-1546.

Wu X-M, Yu Y, Han L-B, Li C-L, Wang H-Y, Zhong N-Q, Yao Y, Xia G-X (2012) The Tobacco BLADE-ON-PETIOLE2 Gene Mediates Differentiation of the Corolla Abscission Zone by Controlling Longitudinal Cell Expansion. Plant Physiol 159: 835-850.

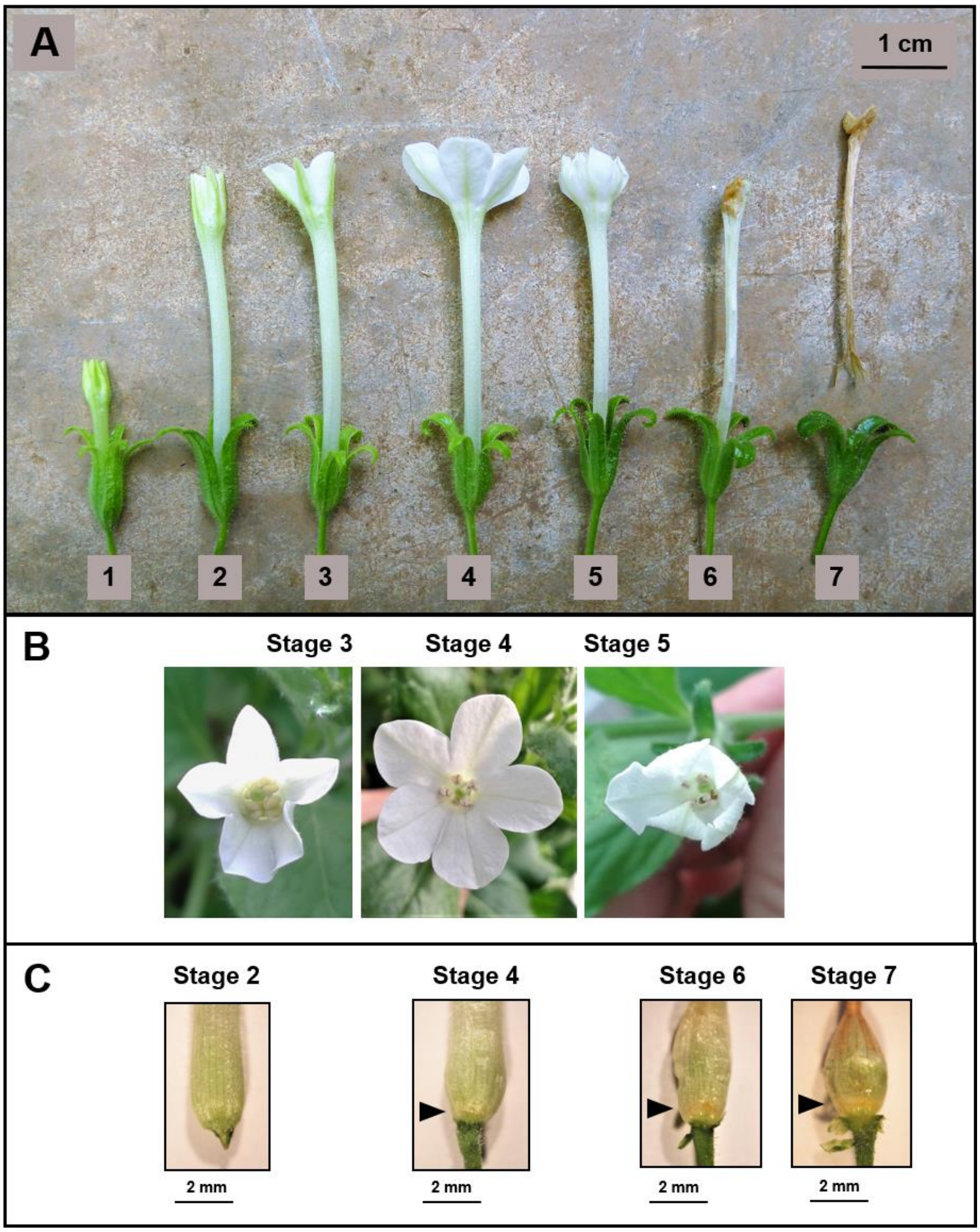

Figure 2. Developmental stages of the flower of *Nicotiana benthamiana*. **(A)** The corolla life span is divided in seven stages which include elongation of the corolla tube [stages 1 and 2], corolla opening [stages 3 and 4] and corolla collapse and senescence [stages 5, 6 and 7]. **(B)** Dehiscence of the five anthers occurs during the opening of the corolla between stages 3 and 4 and are completely dehiscent by stage 5 when corolla limb lobes curl inwards. **(C)** The basal part of the corolla tube is swollen and turgid in stage 2 while it begins to lose turgidity in stage 4. A brown ring appears at the base of the corolla tube at this stage and is clearly visible at the base of the senescent corolla at stage 6 (see arrowheads). The base of the corolla tube begins to lose its structural integrity at stage 7 which allows the corolla to be separated from the flower receptacle by a gentle pulling out.
