# Supplementary material for: IDA (INFLORESCENCE DEFICIENT IN ABSCISSION)-like peptides and HAE (HAESA)-like receptors regulate corolla abscission in Nicotiana benthamiana flowers
Source: BMC Plant Biol. 2021 May 21;21:226. doi: 10.1186/s12870-021-02994-8 (PMC8139003; doi:10.1186/s12870-021-02994-8)
Supplement: Supplementary file 6 — Additional file 6. Primers used in this work. [file 12870_2021_2994_MOESM6_ESM.pdf]

Additional File 6

Primers used in this work.

**Primer name**	**Sequence (5’ → 3’)**
Silencing trigger gene fragment of *NbenIDA1* homeologs	
JG14D	CAAGAATATTTTCAACACAACATTTGAAGG
JG15R	CCACAAAAGCATTGTGTCTCTTAGATGGAGC
Silencing trigger gene fragment of *NbenHAE* homeologs	
JGHAED	CTTGAGTCACTCAATTTATTTGAGAA
JGHAER	GGAAATTTACCCGAAAATTTGTTGTATGAAACGTC
Coding sequence of *NbenIDA1A*	
IDABF	ATGGCCTCCTCCTCCTCCTCTTCC
IDABR	TCAATTTTGAGGAGAAGAGTCCACAAAAGC
Coding sequence of *CitIDA3*	
JG16	ATGGCTTCTTCTTCTTCTTCTTCTTCTTCTAAG
JG17	TCAATTTTGAGTAGAATCCACAACAGAATTG
Coding sequence of *AtIDA*	
AtIDA	ATGGCTCCGTGTCGTACGATG
AtIDAR	TCAATGAGGAAGAGAGTTAACAAAAGAGTTG
Overexpression of *NbenIDA1A*	
NbenIDA1A_F	TGAAGCAAGACCAGGAAGAATG
NbenIDA1A_R	GGAACCCCTTTTGGTAGCATAG
NbenPP2A_F	GACCCTGATGTTGATGTTCGCT
NbenPP2A_R	GAGGGATTTGAAGAGAGATTTC
Flanking 30PmlI	
KU17L	ATGTAACTTCAAGTCCACTGTACAATCGTGGG
KU7L	ATCTTGGATTCAGATTCATGAGGCTCCG

All primers except those used to confirm the systemic infection and the overexpression of *NbenIDA1A* had their 5' end phosphorylated.
